# Medicinal Plants for a Healthy Gut Microbiome: Scientific Insights into Modern Herbal Applications

**DOI:** 10.3390/ijms262210875

**Published:** 2025-11-09

**Authors:** Katarzyna Pacyga, Aleksandra Tabiś, Paweł Pacyga

**Affiliations:** 1 Department of Environment Hygiene and Animal Welfare, Faculty of Biology and Animal Science, Wrocław University of Environmental and Life Sciences, 50-375 Wrocław, Poland; 2 Department of Food Hygiene and Consumer Health Protection, Wrocław University of Environmental and Life Sciences, 50-375 Wrocław, Poland; aleksandra.tabis@upwr.edu.pl; 3 Department of Thermodynamics and Renewable Energy Sources, Faculty of Mechanical and Power Engineering, Wrocław University of Science and Technology, 50-370 Wrocław, Poland; pawel.pacyga@pwr.edu.pl

**Keywords:** medicinal plants, healthy gut microbiome, dysbiosis, health disorders, anti-inflammatory activity, antioxidant activity, antimicrobial activity, prebiotic effect

## Abstract

The human gut microbiome is a complex ecosystem of microorganisms fundamental to human health, influencing metabolism, immunity, and neurological function. Dysbiosis, or an imbalance in this microbial community, is increasingly linked to a range of chronic diseases, from inflammatory bowel disease to metabolic syndrome. This article explores the therapeutic potential of several common botanicals in modulating the gut microbiota and promoting intestinal health. We delve into the phytochemical composition and pharmacological properties of nine medicinal plants: globe artichoke, aloe vera, German chamomile, pot marigold, Ceylon cinnamon, dandelion, fennel, garlic, ginger, and green tea. We focus on their anti-inflammatory, antioxidant, antimicrobial, and prebiotic effects. The article also discusses the scientific evidence supporting their use, acknowledges the limitations of current research, and highlights considerations for safe and effective application. We conclude by summarising the significant role of these herbal remedies in modern complementary medicine and proposing future research directions to further elucidate their mechanisms of action and optimise their use for gut health.

## 1. Introduction

The human gut microbiome is a complex and dynamic ecosystem comprising trillions of microorganisms, including bacteria, viruses, fungi, and archaea, primarily residing in the gastrointestinal tract, and plays a pivotal role in maintaining physiological homeostasis and overall health [1,2]. Increasing evidence has underscored that the gut microbiome is essential for various physiological processes, including: gut barrier function [3], immune system development [4,5], metabolic processes [6], and neuroendocrine system [7]. Disruptions to this intricate microbial balance, commonly referred to as dysbiosis, have been implicated in a wide range of disorders, from inflammatory bowel disease and obesity [8] to neurodegenerative [9] and mood disorders [10]. The composition and function of the gut microbiome are shaped by a complex interplay of multiple factors. Among them, diet plays a central role. Specific foods, food groups, and dietary components, such as prebiotics, dietary fibres, and probiotics, can significantly modulate the microbiome, thereby influencing both health and disease outcomes [11,12]. In addition to diet, host genetics and environmental exposures also contribute to microbiome variability, affecting its stability and diversity over time [13,14,15]. Another important set of factors includes age and lifestyle. From birth through adulthood, the gut microbiome undergoes dynamic changes influenced by developmental stage, daily habits, and medical interventions, including antibiotic use [1,11,16].

In response to growing recognition of the microbiome’s centrality in health and disease, there has been a marked surge in scientific and public interest in dietary and phytotherapeutic strategies aimed at modulating gut microbial communities [17,18,19]. Among these, medicinal plants have emerged as promising agents due to their bioactive compounds, which may influence microbial composition and function, either directly or indirectly, through prebiotic-like effects or antimicrobial properties [20,21,22,23].

This review aims to critically evaluate commonly used medicinal plants (globe artichoke, aloe vera, German chamomile, pot marigold, Ceylon cinnamon, dandelion, fennel, garlic, ginger, green tea) in the context of gut microbiome modulation. Emphasis is placed on their phytochemical profiles, mechanisms of action, and evidence from both preclinical and clinical studies concerning their impact on gut microbial composition and associated health outcomes. Through this lens, the paper seeks to bridge traditional herbal knowledge with contemporary microbiome science, offering insights into the therapeutic potential of botanical interventions for gut health.

## 2. The Healthy Gut Microbiome

**Figure 1:**
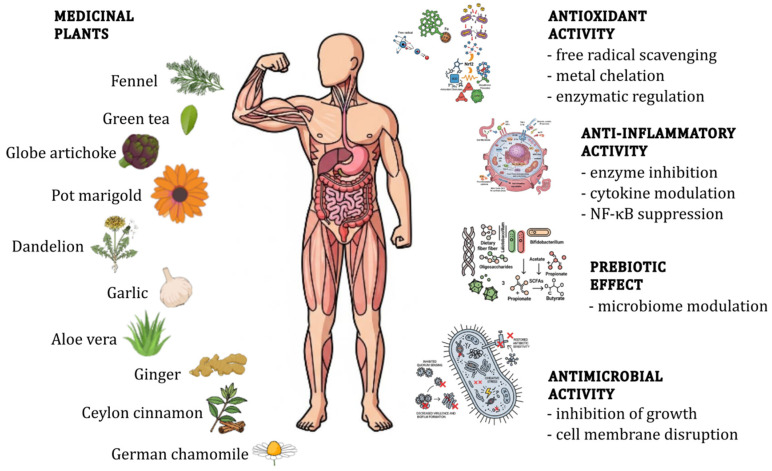
Schematic overview of the activities of medicinal plants.

Characterising the healthy human gut microbiome is inherently challenging due to its dynamic nature and the pronounced inter-individual variability in microbial composition (Figure 1) [24]. Despite these complexities, an emerging consensus defining “a healthy gut microbiome” led to the following conclusions: (1) a single idealised community composition cannot characterise it, (2) it is marked by greater resistance and resilience to disruption, (3) specific microbial patterns may heighten susceptibility to infection and disease, and (4) it remains unclear whether dysbiosis—an imbalance in microbial communities—is a cause or consequence of disease [25,26,27]. This intricate community, comprising trillions of microorganisms, including bacteria, archaea, fungi, and viruses, plays a pivotal role in modulating host metabolism, bolstering immune function, and safeguarding against pathogenic invasions [28]. A balanced intestinal microbiome is characterised not simply by the composition of particular bacterial species, but by the collective physiological capabilities and biochemical productivity of the complete microbial ecosystem [29,30]. The composition of the microbiome is shaped during the first years of human life and is influenced by the mode of delivery and the type of infant nutrition (formula feeding vs. breastfeeding). Its diversity may be markedly reduced by antibiotic use and dietary restrictions in early childhood [25,26,27]. In breastfed, vaginally delivered infants, the early gut microbiota is predominantly composed of *Bifidobacterium* and *Bacteroides* species, which have co-evolved with humans and are specialised in metabolising human milk oligosaccharides [31]. However, there is insufficient clinical evidence to establish these genera as the standard of a healthy microbiota in the developing child. In adults, a healthy microbiome is stable and adaptable and shaped more by environment than host genetics [27]. It has the capacity to efficiently metabolise dietary fibres, produce essential vitamins, and synthesise short-chain fatty acids that serve as primary energy sources for colonic epithelial cells while providing diverse physiological benefits to the host [30,32,33].

At the phylum level, the human gut microbiome is typically dominated by four bacterial phyla: *Firmicutes*, *Bacteroidetes*, *Actinobacteria*, and *Proteobacteria* [34]. While these phyla are consistently prevalent, their relative proportions can vary significantly depending on factors such as age, diet, genetics, and geographical location [35]. *Firmicutes* and *Bacteroidetes* generally constitute the majority of the gut microbiota composition [36,37]. The *Firmicutes* phylum is a large and diverse group encompassing a wide range of bacteria with varying metabolic capabilities [38]. Many members of the *Firmicutes* phylum are involved in the fermentation of complex carbohydrates and the production of short-chain fatty acids, which are essential for maintaining gut health and energy homeostasis. *Bacteroidetes* phylum, primarily represented by the genus *Bacteroides*, is also actively involved in carbohydrate metabolism and the breakdown of complex polysaccharides. Notably, while the relative abundance of these phyla may vary considerably among individuals, the core functional roles they perform within the gut ecosystem appear to be largely conserved [24,39]. However, the use of the relative ratio between *Firmicutes* and *Bacteroidetes*, often considered by some researchers as a key indicator of gut health, and linking the proportion between *Firmicutes* and *Bacteroidetes* with metabolic disorders appears to be an oversimplification. Research conducted by Magne et al. [40] demonstrates that the relative abundance of *Firmicutes* and *Bacteroidetes* phyla is highly variable among individuals from the same population. This likely results from multiple lifestyle-related factors, including diet, physical activity, food additives and contaminants, antibiotic consumption, physical activity, and others that influence gut microbiota composition. Evidence suggesting an association between obesity and changes in the *Firmicutes*/*Bacteroidetes* ratio is not convincing and requires improved characterisation of study subjects and clear identification of covariates that may affect microbiota composition and confound result interpretation [40]. The gut microbiota is known to have a symbiotic relationship with its host [41]. The gut microbiota acts like a “superorganism” inside the human host and aids in the assimilation of food, produces metabolites that nourish the host, protects the host from infection, maintains the function and morphology of intestinal epithelial cells, and regulates host immunity [42,43].

The intestinal barrier is primarily maintained by the apical junctional complex, consisting of tight junctions (TJs) and adherens junctions that regulate intestinal permeability and interactions with gut microbiota. Tight junctions are composed of structural proteins such as claudins, occludin, junctional adhesion molecule-A (JAM-A), and intracellular scaffolding proteins including zonula occludens (ZOs) and cingulin [44]. Cytokines like interleukin-13 (IL-13) and tumour necrosis factor-alpha (TNF-α) modulate TJ integrity by activating signalling pathways such as myosin light chain kinase (MLCK) and inducing claudin-2 expression, leading to increased permeability [44,45]. Inflammatory responses and microbial toxins, including those from *Clostridium difficile* and *Salmonella enterica*, further disrupt TJ proteins, promoting epithelial leakiness. Conversely, probiotics such as *Lactobacillus rhamnosus* GG and *L. reuteri* D8 enhance occludin and ZO-1 expression, improving barrier integrity [46]. Regulation of TJ proteins involves multiple signalling cascades, notably the mitogen-activated protein kinases (MAPK) and nuclear factor kappa B (NF-κB) pathways, which respond to inflammatory stimuli and microbial interactions. NF-κB, a central regulator of inflammation and immune response, modulates TJ protein expression and intestinal permeability. Inhibition of NF-κB activation, as demonstrated for certain plant-derived compounds like kaempferol glycosides in STW 5-II, can restore epithelial integrity and reduce inflammation. Overall, dietary components and phytochemicals may maintain intestinal barrier function by modulating gut microbiota composition and attenuating NF-κB/MAPK-mediated inflammatory signalling that disrupts tight junction assembly [47].

Beyond its localised functions within the gastrointestinal tract, the gut microbiome profoundly influences systemic health through intricate bidirectional communication pathways involving neural, endocrine, and immunological signalling [48]. Gut microbiota produces metabolites which in turn promote gut health by participating in metabolic pathways, regulating gene expression, and synthesising beneficial bioactive compounds [30,49]. The production of essential amino acids and vitamins like vitamin K, thiamine, folate, biotin, riboflavin and pantothenic acid also maintains gut health [32]. Maintaining a healthy gut microbiome necessitates a multifaceted approach, encompassing dietary modifications, lifestyle interventions, and, in some cases, targeted therapeutic interventions aimed at restoring microbial balance and promoting overall host well-being [32].

The term prebiotics was first defined in 1995 as “non-digestible food ingredients that confer health benefits to the host by selectively promoting the growth and activity of specific bacterial populations in the colon, thereby enhancing host well-being” [50]. In general, non-digestible carbohydrates are regarded as prebiotics. To be classified as such, they must meet specific criteria: (i) resistance to digestion by mammalian enzymes and gastric acidity, (ii) susceptibility to fermentation by gut microbiota, and (iii) the ability to enhance the growth and activity of beneficial microorganisms [25,27]. Traditionally, inulin, galacto-oligosaccharides (GOS), and fructo-oligosaccharides (FOS) have been recognised as the primary representatives. More recently, a broader range of dietary fibres and other compounds have been identified as potential prebiotics, each contributing various health-promoting effects. Based on current evidence, eight categories of prebiotic nutritional fibres have been described as beneficial to human health (Table 1) [51].

## 3. Microbiome Imbalance and Disease

**Figure 2:**
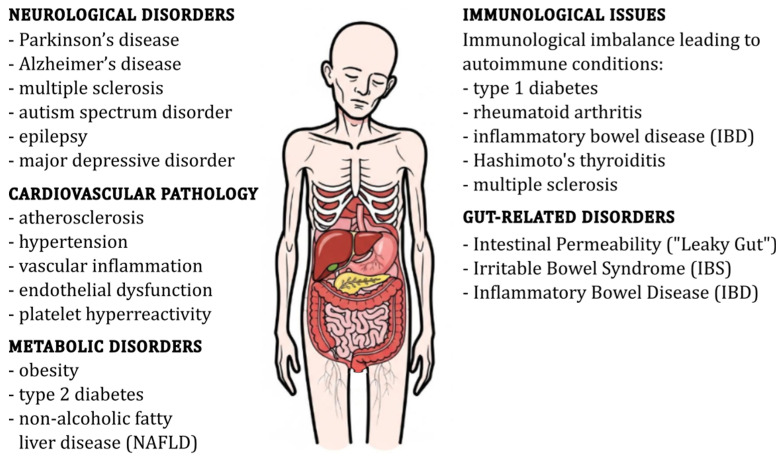
Schematic representation of gut dysbiosis and its associated health disorders.

Gut dysbiosis, defined as a compositional and functional deviation of the intestinal microbiota from a health-compatible configuration, has been increasingly recognised as a key factor in the aetiology of a wide range of chronic diseases (Figure 2). It is typically marked by a decline in microbial diversity, an enrichment of facultative anaerobes or pathobionts, and a depletion of taxa with anti-inflammatory or trophic capacities [29,65], such as *Faecalibacterium prausnitzii* (production of microbial anti-inflammatory molecules (MAMs); modulation of bile acid metabolism and farnesoid X receptor (FXR) signalling pathways; maintenance of gut health by promoting intestinal mucosal repair and inhibiting inflammation) [66], *Akkermansia muciniphila* (crucial for maintaining the gut barrier; linked to improved metabolic health; plays a role in immune regulation) [67], *Bifidobacterium* spp. (promoting gut health and modulating the immune system; regulation of intestinal flora, control of glycolipid metabolism; anti-colitis effects) [68], *Roseburia* spp. (butyrate-producing bacteria that support gut health) [69], and other butyrate-producing *Firmicutes* (production of butyrate, a short-chain fatty acid essential for colon health and anti-inflammatory effects) [70].

Beyond taxonomic shifts, dysbiosis entails profound functional reprogramming of the microbiome, including reduced expression of genes involved in short-chain fatty acid (SCFA) biosynthesis, tryptophan catabolism, and oxidative stress resistance [71,72]. One of the principal metabolic consequences of dysbiosis is impaired fermentation of dietary polysaccharides, resulting in diminished production of SCFAs—notably butyrate, propionate, and acetate. These metabolites serve not only as vital energy sources for colonocytes but also exert pleiotropic effects including the regulation of immune homeostasis, enhancement of epithelial repair and intestinal barrier integrity, modulation of luminal oxygen tension, attenuation of inflammatory signalling pathways, and systemic control of glucose and lipid metabolism [73,74,75,76]. Butyrate, in particular, inhibits histone deacetylase, which leads to changes in gene expression. This inhibition affects a wide range of genes involved in inflammatory responses, including those encoding NF-κB and other pro-inflammatory cytokines [77]. Moreover, butyrate promotes the expansion of *Foxp3^+^* regulatory T cells (Tregs), which play a crucial role in maintaining immune homeostasis and suppressing inflammatory responses. This effect is mediated through histone deacetylase (HDAC) inhibition, which enhances the expression of Treg-associated genes like *FoxP3* while suppressing pro-inflammatory T helper 17 (Th17) cells [78].

A decline in SCFA-producing taxa, such as those from the genera *Prevotella* and *Butyricimonas*, is closely linked with increased intestinal permeability [79], a phenomenon often referred to as “leaky gut,” whereby compromised barrier integrity permits the translocation of microbe-associated molecular patterns (MAMPs) from the gut lumen into the lamina propria and potentially into systemic circulation [80,81,82,83]. Lipopolysaccharide (LPS), a component of the outer membrane of Gram-negative bacteria, is a well-documented MAMP that can translocate across a compromised intestinal barrier. Increased levels of LPS in the bloodstream are associated with systemic inflammation and various diseases, including metabolic dysfunction-associated steatotic liver disease (MASLD) and alcoholic liver disease (ALD) [82,84]. Similar to LPS, other MAMPs such as flagellin and peptidoglycan can also translocate through a dysfunctional barrier. These molecules activate the immune system via pattern recognition receptors (PRRs), contributing to inflammation and disease progression [83]. The translocation of these MAMPs can occur through paracellular routes, which are regulated by tight junctions. Disruption of tight junctions increases paracellular permeability, facilitating the movement of MAMPs into the lamina propria and beyond [80,81,85]. Once in the systemic circulation, these MAMPs can trigger widespread immune responses, leading to conditions such as systemic inflammatory response syndrome (SIRS) and contributing to the pathogenesis of various diseases [80,81,82,83]. These molecules engage PRRs, including toll-like receptors (TLRs) [86,87] and NOD-like receptors (NLRs) [88,89], which typically leads to the activation of the MyD88-dependent pathway, which subsequently activates NF-κB, a key transcription factor in the inflammatory response [86,89,90]. NLRs can form inflammasomes, which are multiprotein complexes that activate inflammatory responses, including the production of pro-inflammatory cytokines such as IL-1β, IL-6, IL-8, and TNF-α [89,90,91]. This inflammatory response is crucial for pathogen elimination but can lead to chronic low-grade inflammation if dysregulated [89,92]. Chronic low-grade inflammation, or metaflammation, has been implicated in the pathophysiology of several disorders, including metabolic, autoimmune, and neuropsychiatric disorders [89].

A characteristic dysbiotic signature in inflammatory bowel disease (IBD) involves the expansion of *Proteobacteria* alongside a reduction in the abundance of bacterial members from the *Bacteroidetes* and *Firmicutes* phyla [93]. This microbial imbalance drives aberrant mucosal immune activation, sustained cytokine release, and polarisation towards pro-inflammatory T helper subsets, including Th1 and Th17 cells [94]. The resulting inflammatory milieu perturbs the anaerobic gut environment through mucosal oxygenation, thereby favouring facultative anaerobes such as *Escherichia coli* and *Enterococcus faecalis*, which further exacerbate dysbiosis and epithelial damage [95,96].

Irritable bowel syndrome (IBS), although classified as a functional gastrointestinal disorder, also exhibits microbial alterations. These include reduced microbial diversity [97,98,99] and an overrepresentation of pro-inflammatory or gas-producing taxa [100], contributing to visceral hypersensitivity [101,102], altered motility [102], and mucosal immune dysregulation [100,101,102]. Communication between the gut microbiota and the central nervous system via neuroimmune, neuroendocrine, and vagal pathways—the gut–brain axis—plays a critical role in IBS symptomatology [101,102].

Beyond the gastrointestinal tract, dysbiosis is increasingly recognised as a contributor to various metabolic disorders, including type 2 diabetes, obesity, and metabolic syndrome [103,104,105]. The translocation of LPS from the gut into the circulation is a significant contributor to adipose tissue inflammation and systemic insulin resistance. LPS activates TLR4 on immune cells, leading to the release of pro-inflammatory cytokines and subsequent impairment of insulin signalling. The activation of TLR4 by LPS not only fosters insulin resistance but also contributes to hepatic steatosis (fatty liver disease) [106,107,108,109]. Bile acids, which are metabolised by gut bacteria, play a crucial role in regulating host metabolism. Dysbiosis can alter bile acid profiles, impacting lipid metabolism, glucose homeostasis, and inflammatory responses. Bile acids activate FXR and G protein-coupled bile acid receptor (TGR5), which are involved in regulating glucose and lipid metabolism, as well as inflammatory responses. Alterations in bile acid signalling pathways due to dysbiosis can thus influence metabolic health [110,111,112,113,114,115].

Non-alcoholic fatty liver disease (NAFLD) and its progressive form, non-alcoholic steatohepatitis (NASH), exemplify hepatic conditions linked to microbial imbalance. Increased gut permeability facilitates translocation of microbial products, activating hepatic Kupffer cells and promoting chronic liver inflammation and fibrosis [116,117,118,119,120].

The gut microbiota also engages in bidirectional communication with the central nervous system, modulating neurological function through multiple channels, including the nervous system, endocrine system, and immune system [121,122,123]. Dysbiosis can lead to disruptions in tryptophan catabolism, favouring the kynurenine pathway over serotonin synthesis. This shift results in increased production of neuroactive and potentially neurotoxic metabolites such as quinolinic acid, which are implicated in neuroinflammation and neurological dysfunction [124,125,126,127]. Reduced central serotonin levels and elevated kynurenine/tryptophan ratios have been reported in patients with depression and Alzheimer’s disease, supporting the concept of inflammation-driven tryptophan metabolic imbalance [127,128]. Peripheral immune activation and systemic inflammation further potentiate kynurenine pathway activity, leading to the accumulation of neurotoxic metabolites and activation of microglia. These immune-mediated processes impair blood–brain barrier integrity and contribute to sustained neuroinflammation, which plays a central role in the pathogenesis of neuropsychiatric and neurodegenerative disorders [125,126,129]. In parallel, microbial components such as amyloids and lipopolysaccharides can directly trigger microglial activation and compromise blood–brain barrier (BBB) function. LPS, a component of the outer membrane of Gram-negative bacteria, can activate microglia via Toll-like receptor 4 signalling, leading to neuroinflammation and BBB dysfunction [130,131]. LPS-activated microglia produce reactive oxygen species through nicotinamide adenine dinucleotide phosphate (NADPH) oxidase, which disrupts tight junction proteins in the BBB, increasing its permeability [130,132]. Bacterial amyloids likewise contribute to neuroinflammation and BBB impairment. Moreover, amyloid-β (Aβ) peptides, associated with Alzheimer’s disease, can form plaques in conjunction with bacterial components such as LPS, further amplifying microglial activation and inflammatory processes [133,134]. These mechanisms have been implicated not only in Parkinson’s [134] and Alzheimer’s disease [135,136,137], but also in multiple sclerosis [134,138], autism spectrum disorder [138], epilepsy [138], and major depressive disorder [138], where chronic neuroinflammation and altered gut–brain signalling are increasingly recognised as key contributors to disease progression. Causal roles have been supported by faecal microbiota transplantation studies, wherein microbiota from affected individuals induced disease-relevant behavioural and neurochemical changes in germ-free animals. Several studies have demonstrated this phenomenon across various neuropsychiatric and neurodevelopmental disorders [139,140,141,142].

Autoimmune conditions also demonstrate microbiota-associated dysregulation. In rheumatoid arthritis (RA), increased abundance of *Prevotella copri* may drive systemic inflammation through mechanisms such as molecular mimicry, where bacterial antigens resemble host proteins, leading to immune system confusion and activation [143,144,145]. In multiple sclerosis (MS), reduced microbial diversity and enrichment of pro-inflammatory taxa contribute to peripheral immune dysregulation and blood–brain barrier disruption [146,147,148]. Moreover, early-life microbial disturbances are linked to heightened risk of atopic diseases such as allergic rhinitis and asthma [149,150,151]. Reduced production of SCFAs like butyrate and propionate can lead to a decrease in Treg populations, which are essential for suppressing Th2 responses. Without sufficient Tregs, the immune system may become skewed towards a Th2-dominant profile, which is associated with allergic responses and impaired mucosal tolerance [152,153,154].

Beyond autoimmune and neurological conditions, the cardiovascular system is similarly influenced by gut microbial metabolites. Specific taxa metabolise dietary choline, L-carnitine, and phosphatidylcholine into trimethylamine (TMA), which is converted in the liver to trimethylamine N-oxide (TMAO). Elevated TMAO levels are strongly associated with increased atherosclerosis, platelet hyperreactivity, inflammation and endothelial dysfunction, illustrating a critical link between microbial metabolism and vascular health [155,156,157,158].

At the molecular level, dysbiosis disrupts epithelial and systemic immune homeostasis through diverse mechanisms. Reduced availability of SCFAs impairs tight junction protein expression (e.g., occludin, claudins), thereby weakening barrier integrity [159]. Concurrently, altered microbial metabolism affects the production of indole derivatives, natural ligands of the aryl hydrocarbon receptor (AhR), with downstream effects on mucosal immunity and epithelial differentiation [160,161]. Microbial transformation of primary into secondary bile acids occurs through various enzymatic modifications, including deconjugation, dehydroxylation, and epimerization. This process significantly alters the composition of the bile acid pool, thereby influencing the host’s intestinal mucosal immunity and overall health [162,163,164]. Secondary bile acids interact with nuclear and membrane-bound receptors, such as FXR and TGR5, modulating both inflammation and gut physiology. While they can attenuate FXR activation, loss of microbe-derived bile acids during inflammation may lead to increased FXR activity, which is associated with exacerbation of conditions like graft-versus-host disease (GVHD) [165]. Conversely, TGR5 activation by secondary bile acids promotes a tolerogenic immune phenotype in the intestine, reducing local inflammation [163]. In addition to their immunomodulatory roles, bile acids also regulate gut motility by acting on FXR and TGR5 receptors, both of which influence gastrointestinal function and transit [163,166].

Specific microbial patterns, such as elevated levels of *Ruminococcus gnavus* have been observed in IBD patients, suggesting its potential as a biomarker for this condition. Additionally, *R. gnavus* is linked to metabolic disorders, further supporting its role as a biomarker [167,168,169]. Moreover, can foster horizontal gene transfer (HGT), which facilitates the emergence of antibiotic resistance genes (ARGs) [170,171,172]. This process is particularly concerning in inflamed environments where oxidative stress increases mutagenesis and DNA damage, further enhancing the potential for HGT [170].

While most studies have focused on bacterial communities, it is increasingly evident that the gut mycobiome and virome also shape host–microbiota interactions [173,174]. Overgrowth of *Candida* spp. and alterations in bacteriophage populations have been reported in IBD, with potential immunostimulatory and regulatory consequences [175,176,177]. Moreover, host genetic polymorphisms, such as those in nucleotide-binding oligomerisation domain-containing protein 2 (NOD2) and *ATG16L1*, further modulate microbial configuration and susceptibility to chronic inflammatory states [178,179,180,181].

Taken together, these insights underscore the far-reaching consequences of gut microbial imbalance across multiple physiological systems. Although many observed associations remain correlative, converging evidence from metagenomic studies, gnotobiotic models, and faecal microbiota transplantation strongly supports a causal role in numerous pathologies. These findings provide a compelling rationale for therapeutic strategies aimed at restoring microbial equilibrium. As elaborated in the following sections, medicinal plants offer a versatile and underexplored platform for microbiome modulation, with promising implications for integrative, microbiota-targeted interventions in human health.

## 4. Common Medicinal Plants with Documented Microbiome Effects

This section provides a focused overview of ten medicinal plants selected for their traditional use and contemporary scientific relevance. Each plant is discussed with respect to its botanical characteristics, phytochemical composition, and documented antioxidant, anti-inflammatory, antimicrobial activities, all of which may contribute to gut microbial balance and overall gastrointestinal health.

### 4.1. Globe Artichoke (Cynara scolymus L.)

**Figure 3:**
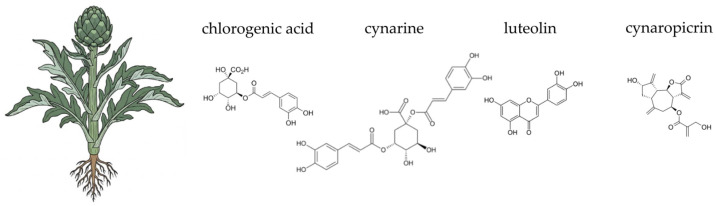
Botanical illustration of globe artichoke and the chemical structures of selected four key bioactive compounds characteristic for this plant.

*Cynara scolymus* L., commonly known as the globe artichoke, is a perennial herbaceous plant belonging to the *Asteraceae* family (Figure 3). Native to the Mediterranean region, it has a long history of both culinary and medicinal use, particularly valued for its supportive effects on liver and digestive function [182]. Traditionally consumed as a herbal infusion or foodstuff, artichoke is now widely available in the form of standardised leaf extract supplements [183,184,185]. The edible parts of the plant, including immature flower heads and leaves, are rich in a broad spectrum of bioactive compounds. The plant is generally considered safe when consumed in customary dietary amounts; however, caution is advised in individuals with biliary obstruction or known hypersensitivity to other members of the *Asteraceae* family [184]. The complex phytochemical profile of *C. scolymus* underpins its traditional and modern applications in dietary and therapeutic contexts.

The globe artichoke contains a diverse array of phytochemicals, including phenolic acids, flavonoids, sesquiterpene lactones, and inulin-type fructans. Key hydroxycinnamic acid derivatives, such as cynarin (1,5-dicaffeoylquinic acid) and chlorogenic acid, are abundant and well-studied for their hepatoprotective and choleretic properties [185,186,187]. Flavonoids like luteolin and apigenin also contribute significantly to the plant’s pharmacological potential [185,187,188,189]. Artichoke leaves also contain sesquiterpene lactones, particularly cynaropicrin, which is associated with its characteristic bitter taste [188,190,191]. Crucially for gut health, the edible parts of the plant contain inulin, a non-digestible carbohydrate with recognised prebiotic properties [185,192]. The complex phytochemical profile of *C. scolymus* underpins its traditional and modern applications in dietary and therapeutic contexts.

The antioxidant activity of *Cynara scolymus* is primarily attributed to its high content of polyphenolic compounds. These compounds, including cynarin and chlorogenic acid, mitigate oxidative stress through a multi-faceted approach. First, they act as direct free radical scavengers, neutralising reactive oxygen species (ROS) and preventing oxidative damage to biomolecules [183,193,194,195,196]. Second, they can chelate transition metal ions like Fe^2+^ and Cu^2+^, which prevents the formation of highly reactive hydroxyl radicals [197,198]. Third, these phytochemicals modulate endogenous antioxidant defences by activating the nuclear factor erythroid 2-related factor 2 (Nrf2) signalling pathway, which upregulates enzymes such as superoxide dismutase (SOD) and glutathione peroxidase (GPx) [183,199]. In the gut, oxidative stress is a key factor in damaging the intestinal barrier and promoting mucosal inflammation. Artichoke-derived polyphenols, such as chlorogenic acid and dicaffeoylquinic acids, help protect intestinal epithelial cells by attenuating ROS-mediated damage and preserving tight junction proteins like claudin-1 and occluding structure [200,201,202]. Furthermore, due to their limited absorption in the upper gastrointestinal tract, these polyphenols reach the colon, where they undergo extensive microbial biotransformation. The gut microbiota metabolises them into smaller, more bioavailable compounds, such as caffeic acid, which possess enhanced antioxidant properties. This process directly links the antioxidant efficacy of *C. scolymus* to a healthy host-microbe relationship [203,204,205].

Beyond its antioxidant properties, *Cynara scolymus* exhibits notable anti-inflammatory effects. These are closely associated with its polyphenolic constituents, which modulate key inflammatory pathways. One central mechanism involves the inhibition of the NF-κB signalling cascade, a critical regulator of pro-inflammatory gene expression. Artichoke-derived compounds, such as cynarin and luteolin, prevent NF-κB translocation to the nucleus, thereby suppressing the transcription of inflammatory mediators including TNF-α and IL-6 [206,207]. Studies have also shown that artichoke leaf extracts can reduce inflammation in models of colitis by decreasing pro-inflammatory cytokines [183,208]. The downregulation of inducible nitric oxide synthase (iNOS) and modulation of MAPK pathways further contribute to the anti-inflammatory and immunomodulatory role of *C. scolymus* [207,209,210]. At the intestinal level, chronic low-grade inflammation disrupts mucosal homeostasis. By attenuating pro-inflammatory cytokine release and limiting oxidative signalling, artichoke polyphenols help preserve epithelial barrier function and promote a tolerogenic immune environment in the gut. This is particularly relevant given the role of inflammation in gastrointestinal disorders like inflammatory bowel disease [205,211,212,213,214]. The anti-inflammatory effects of *C. scolymus* contribute to a favourable gut environment, which is essential for maintaining a balanced and healthy microbiota.

Additionally, globe artichoke is well-known for its choleretic (bile-stimulating) and cholagogue (bile-releasing) effects, mediated by compounds like cynarin and cynaropicrin. These actions improve the digestion and absorption of fats and fat-soluble vitamins, which is vital for overall health. By enhancing bile flow, artichoke helps alleviate symptoms of indigestion, such as bloating and stomach cramps, and contributes to a smoother digestive process. This dual action—promoting a healthy microbiome through prebiotic fibre and optimising digestion through bile regulation—makes globe artichoke a powerful ally for a healthy gut [183,184,215].

Multiple studies have demonstrated the antimicrobial properties of artichoke extracts by evaluating their effects against a range of microbial species. The majority of these findings are derived from in vitro investigations. In in vitro studies, researchers tested various bacterial strains, yeasts, and moulds [182]. The antimicrobial properties of artichoke are primarily attributed to bioactive compounds such as chlorogenic acid, cynarin, luteolin, and apigenin [216]. The mechanism of action of these phytochemicals is multifaceted, encompassing disruption of the microbial cell membrane integrity. Moreover, phenolic compounds and flavonoids exhibit the capacity to bind to essential metabolic enzymes within microbial cells, thereby inhibiting their catalytic activity and impairing fundamental physiological processes. Notably, luteolin has been reported to interfere with nucleic acid synthesis by disrupting both DNA replication and RNA transcription, ultimately inhibiting bacterial proliferation [217]. Artichoke effectively inhibits the growth of multiple bacterial strains. Depending on the solvent used for extraction, differences in antimicrobial activity were observed, which can be attributed to variations in the composition of active compounds. The 97% ethanol extract proved to be the most effective against *Escherichia coli* and *Listeria innocua*, showing a high content of active phenolic compounds. In contrast, *Pseudomonas aeruginosa* and *Staphylococcus aureus* were inhibited by the 75% ethanol extract, which was characterised by a high concentration of both phenolics and flavonoids [218]. Fratianni et al. [219] also reported that artichoke bract extracts displayed antibacterial activity against *P. aeruginosa*, *E. coli*, and *Bacillus cereus*. Bound phenolic compounds from artichoke hearts and bracts showed maximum inhibitory effects against *E. coli*, *Bacillus subtilis*, and *S. aureus*, and moderate activity against *P. aeruginosa*, surpassing that of the free phenolic extracts [220]. Vamanu et al. [218] tested artichoke extracts against 15 different microorganisms and found antimicrobial activity against all tested strains. Similarly, studies by Emanuel et al. [218] and Scavo et al. [221] demonstrated that ethanolic extracts of *Cynara cardunculus* var. *scolymus* L. leaves exhibited antibacterial activity against *E. coli*, *Listeria innocua*, *B. cereus*, *S. aureus*, and *P. aeruginosa*, with minimum inhibitory concentrations (MICs) ranging from 5.0 to 15.0 mg·mL^−1^. Alghazeer et al. [222] also confirmed the antibacterial properties of globe artichoke extracts.

The component of artichoke that causes the prebiotic effect is considered to be long-chain inulin-type fructans. The in vitro study revealed that the addition of artichoke extract to ABY and ABT yoghurt significantly increased the viability of *L. acidophilus* LA-5 and *B. lactis* BB-12 at the end of fermentation and during refrigerated storage. Therefore, probiotic herbal yoghurts are more functional from a probiotic viability point of view. Clinical studies conducted by Costabile et al. [223] involving the administration of long-chain inulin extracted from artichoke heads to healthy adults for 3 weeks showed an increase in *Bifidobacteria*, *Lactobacillus* and *Bacteroides—Prevotella* bacteria compared to placebo. No increase in faecal SCAFA was demonstrated.

Several studies and meta-analyses have provided evidence supporting the efficacy of artichoke in lowering hyperlipidemia, blood pressure, and hypertension following its consumption [182]. Inulin from artichoke heads improves lipid metabolism by stimulating cholesterol conversion into bile salts, thereby lowering serum VLDL and LDL-C levels [224,225]. The beneficial effects of artichoke on diabetes mellitus have been demonstrated in numerous studies, primarily attributed to its hypoglycemic properties [182]. An 8-week intervention with twice-daily oral artichoke extract in overweight individuals with IFG significantly reduced blood glucose, insulin levels, HOMA index, and A1c-derived average glucose (ADAG) [224,226]. Daily consumption of both artichoke leaves and heads for 8 weeks in patients with NAFLD in clinical studies resulted in a reduction in serum levels of ALT, AST, triglycerides (TG), total cholesterol (TC), HDL-C, and LDL-C. Following the intake of artichoke head extract, a decrease in vessel diameter, liver size, and serum bilirubin concentration was also observed [227,228].

A study by Fogacci et al. [229] demonstrated that dietary supplementation with polyphenolic fractions from both dry artichoke and bergamot extracts in 90 patients safely led to significant improvements in serum lipid levels, systemic inflammation, NAFLD markers, and endothelial reactivity in otherwise healthy individuals with suboptimal cholesterol levels [229].

### 4.2. Aloe vera (Aloe vera (L.) Burm. f.)

**Figure 4:**
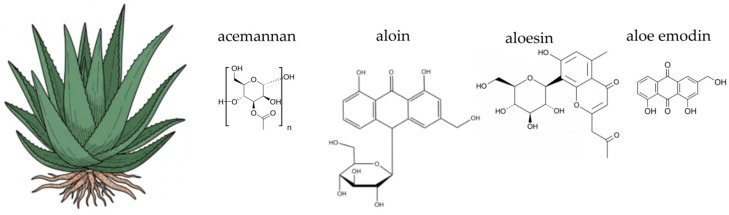
Botanical illustration of aloe vera and the chemical structures of selected four key bioactive compounds characteristic for this plant.

*Aloe vera* (L.) Burm. f., a perennial succulent of the *Asphodelaceae* family, is native to the Arabian Peninsula but is now cultivated globally in arid and semi-arid climates (Figure 4). This plant boasts a long history of use in both folk medicine and as a food source, particularly in supporting digestive health and gut integrity. Traditionally, the fresh inner leaf gel or juice was used, but today, aloe vera is available commercially in various processed forms, including standardised extracts and dietary supplements [230,231,232,233,234]. Beyond its recognised gastrointestinal benefits, aloe vera has been extensively studied for its role in skin health, wound healing, and immune function, and is also investigated for its impact on metabolic regulation and inflammation-related conditions [235,236,237]. The plant’s therapeutic efficacy is underpinned by its rich and complex phytochemical profile, which includes a unique blend of polysaccharides, phenolic compounds, and other bioactive molecules that act synergistically. While the plant’s gel is generally considered safe for consumption, caution is advised with whole-leaf preparations containing anthraquinone derivatives such as aloin, due to their potent laxative properties and potential for cytotoxicity [230,238]. This distinction between the gel and the whole-leaf latex is crucial for understanding the plant’s safe and effective use. The following sections will delve into the specific chemical components of aloe vera and their scientifically supported activities, particularly their contribution to antioxidant and anti-inflammatory pathways.

Aloe vera is a complex plant known for its rich and varied chemical composition. The gel, which is the clear mucilaginous substance from the inner leaf, is primarily composed of water (around 98–99%) but also contains a wide array of bioactive compounds, including polysaccharides, anthraquinones, phenolic compounds, vitamins, minerals, and amino acids. Polysaccharides are a key component, with acemannan being the most prominent. Acemannan is a β-(1,4)-acetylated polymannose that has been extensively studied for its immunomodulatory and anti-inflammatory properties [235,239]. Other polysaccharides include glucomannan and arabinogalactan, which also contribute to the plant’s biological activity. The outer green layer of the leaf, known as the latex or aloin layer, contains a group of compounds called anthraquinones, particularly aloin A and B. These compounds are responsible for the plant’s laxative effects and are often removed during processing for internal use to avoid adverse gastrointestinal reactions [230,231]. The gel also contains phenolic compounds like aloesin and aloeresin, which are chromone derivatives known for their antioxidant activity [240,241]. Additionally, the plant contains essential vitamins such as C and E, minerals like zinc and selenium, and a range of amino acids, which collectively contribute to its multifaceted therapeutic potential [231]. The diverse phytochemical profile of aloe vera is of particular interest in the context of gut health. Key components, such as acemannan and other polysaccharides, act as non-digestible carbohydrates. These compounds can serve as a substrate for beneficial gut bacteria, making aloe vera a potential prebiotic agent [242]. Additionally, anthraquinones, while known for their laxative effects, may also influence the gut environment, though their use requires careful consideration to avoid adverse effects on the gut lining and microbial balance [243].

The antioxidant activity of *Aloe vera* is attributed to its diverse phytochemicals, which work synergistically to neutralise free radicals and protect against oxidative stress. The primary mechanism involves the direct scavenging of ROS and reactive nitrogen species (RNS) by phenolic compounds and vitamins. Phenolic compounds, such as aloesin and other chromone derivatives, donate hydrogen atoms from their hydroxyl groups to neutralise free radicals, effectively stabilising them and preventing chain reactions of oxidative damage [241,244,245,246]. Furthermore, *Aloe vera* extracts have been shown to enhance the activity of endogenous antioxidant enzymes, such as superoxide dismutase, catalase (CAT), and GPx [247,248]. These enzymes are the body’s first line of defence against oxidative stress. SOD converts superoxide radicals into hydrogen peroxide, which is then broken down into water and oxygen by CAT and GPx. This upregulation of enzymatic antioxidants represents a crucial long-term protective mechanism [247,249]. The presence of vitamin C and vitamin E also contributes significantly, as they act as a classic antioxidant team: vitamin C regenerates the reduced form of vitamin E, allowing it to continue its role in protecting cell membranes from lipid peroxidation [243,250,251]. The antioxidant properties of *Aloe vera* are directly relevant to gut health. Chronic oxidative stress in the gut can damage the intestinal barrier, leading to a “leaky gut” syndrome and dysbiosis. By neutralising free radicals and upregulating endogenous antioxidant enzymes, *Aloe vera* can help protect the gut lining from oxidative damage. This protective effect supports the integrity of the intestinal barrier, which is crucial for maintaining a stable and healthy gut microbiome and preventing the translocation of harmful substances into the bloodstream [243,252].

The anti-inflammatory properties of *Aloe vera* are primarily due to the actions of its polysaccharides and various other compounds that modulate inflammatory pathways at the molecular level. Acemannan, a key polysaccharide, exerts its effects by inhibiting the production of key pro-inflammatory cytokines, such as TNF-α and IL-6. This modulation helps to downregulate the inflammatory response [253,254,255]. Additionally, acemannan can stimulate the release of anti-inflammatory mediators, shifting the immune balance towards resolution rather than chronic inflammation. Specifically, acemannan enhances macrophage M2 polarisation and inhibits M1 polarisation, which is crucial for resolving inflammation [256]. Additionally, acemannan has been reported to activate professional antigen-presenting cells such as macrophages and dendritic cells, further supporting its role in modulating immune responses [257,258]. Another critical mechanism involves the inhibition of enzymes central to the inflammatory cascade. Phytosterols, such as lupeol, have been demonstrated to inhibit cyclooxygenase-2 (COX-2), an enzyme responsible for converting arachidonic acid into pro-inflammatory prostaglandins [259]. Similarly, C-glycosyl chromones, including aloesin, can inhibit prostaglandin synthesis, further contributing to the plant’s anti-inflammatory effects [260,261,262,263]. This dual action, modulating cytokine production and inhibiting key enzymes, provides a comprehensive approach to managing inflammation. The synergy between these compounds makes *Aloe vera* a potent agent in both topical and systemic anti-inflammatory applications [264,265,266,267]. The anti-inflammatory effects of *Aloe vera* play a vital role in supporting a healthy gut. Chronic, low-grade inflammation is a major contributor to intestinal dysbiosis and various gastrointestinal disorders. By modulating pro-inflammatory cytokines and inhibiting key enzymes like COX-2, *Aloe vera* helps to reduce intestinal inflammation. This creates a more favourable environment for beneficial bacteria to thrive while suppressing the growth of pathogenic microbes that are often associated with inflammatory states [243,267,268,269]. This mechanism underscores the plant’s potential as a therapeutic agent for managing conditions like inflammatory bowel disease and supporting overall gut homeostasis [253,270].

The complexity of *Aloe vera*’s composition suggests that its biological activities are likely due to the synergistic action of its many components rather than a single active ingredient [271]. Research has shown that *Aloe vera* is rich in a variety of bioactive constituents, including anthraquinones, dihydroxyanthraquinones (such as aloe-emodin and aloin), and saponins. These compounds are believed to contribute significantly to the plant’s various therapeutic properties, including its demonstrated antimicrobial effects against a range of bacteria, fungi, and even some viruses [272]. The antimicrobial mechanism of emodin against *Escherichia coli* has been proposed by Hamman [231] as an inhibition of active membrane transport. In a study conducted by Nejatzadeh-Barandozi [272], which investigated the antibacterial efficacy of various *Aloe vera* extracts against *S. aureus*, *S. pyogenes*, *P. aeruginosa* and *E. coli*, the acetone extract demonstrated maximal antibacterial activity, outperforming both aqueous and ethanol extracts [272]. The treatment of *Aloe vera* gel in combination with antibiotic therapy has shown promise against *Helicobacter pylori* [273]. Beyond its established antibacterial properties, *Aloe vera* also exhibits antifungal activity. The processed gel has been observed to inhibit the growth of *Candida albicans*. Furthermore, a potent anticandidal effect against *C. albicans*, *C. parapsilosis*, and *C. krusei* has been attributed to a specific 14 kDa protein found within *Aloe vera* gel.

The prebiotic effects of *Aloe vera* can be attributed to its complex carbohydrate content, which serves as a nutrient source for beneficial gut bacteria, promoting their growth and activity [271]. The selective stimulation of beneficial bacteria by *Aloe vera* can lead to a cascade of positive effects on gut health, including improved digestion, enhanced immune function, and protection against pathogenic infections [274]. The main bioactive polymer of *Aloe vera*, acemannan, undergoes structural modifications that affect its physicochemical properties and flow behaviour, potentially influencing its prebiotic activity [275]. These carbohydrates resist digestion in the upper gastrointestinal tract and reach the colon, where they are fermented by bacteria, leading to the production of short-chain fatty acids such as acetate, propionate and butyrate, which provide energy to colonocytes and exert various health-promoting effects [276]. *Aloe vera* gel contains glucomannan, a water-soluble polysaccharide, as its main constituent, along with antimicrobial compounds and antioxidants such as saponins and anthraquinones, which may optimise its prebiotic effects [277]

Aloe vera include relief from constipation, detoxification, digestion promotion, and cytoprotection for peptic ulcers [231,273,278]. Moreover, *Aloe vera* exhibits protective effects on the liver and can significantly reduce concentrations of fasting blood glucose, triglyceride, total cholesterol, and LDL-cholesterol, demonstrating its potential in treating diabetes and improving overall metabolic health [279]. *Aloe vera* preparations have been popular for their laxative effects and their ability to treat skin ailments [271]. *Aloe vera* can also act as a co-therapeutic agent because the inner gel may improve adsorption through retention in the gastric mucosa [273]. These diverse mechanisms contribute to the role of *Aloe vera* in traditional medicine, where it has been used for millennia to address a variety of health conditions [231,234].

Numerous clinical studies investigating the effects of aloe in patients with IBS have shown that supplementation improves quality of life and health, with the following effects reported: a laxative effect in IBS-related constipation, increased intestinal motility, stimulation of mucus secretion, and the release of prostaglandin-like compounds in the colon [280,281].

### 4.3. German Chamomile (Matricaria chamomilla L.)

**Figure 5:**
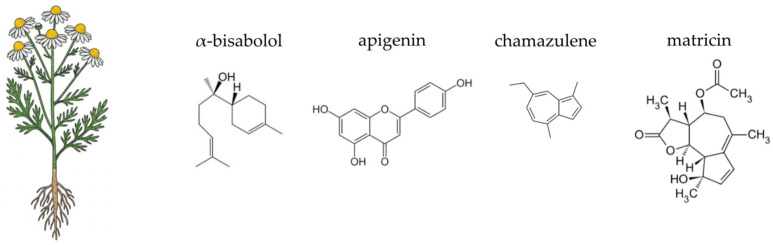
Botanical illustration of German chamomile and the chemical structures of selected four key bioactive compounds characteristic for this plant.

German chamomile (*Matricaria chamomilla* L.), a member of the *Asteraceae* family, is a ubiquitous annual plant with a rich history in traditional European medicine (Figure 5). Widely cultivated and naturalised across Europe and Asia, it has been a cornerstone of herbal remedies for centuries, particularly for its calming, anti-inflammatory, and spasmolytic effects on the gastrointestinal tract [282,283,284]. Chamomile preparations are commonly used to support the treatment of various human ailments, such as allergic rhinitis, inflammatory conditions, muscle cramps, menstrual irregularities, insomnia, peptic ulcers, skin wounds, and gastrointestinal disturbances. In addition, chamomile essential oils are widely applied in the fields of cosmetics and aromatherapy, valued for their soothing and anti-inflammatory properties [283,285,286]. Traditionally consumed as a tea or decoction from its dried flower heads, it is now available commercially in various forms, including standardised extracts, essential oils, and dietary supplements. The plant’s therapeutic efficacy is derived from a complex synergy of its diverse phytochemicals, notably flavonoids, terpenoids, and coumarins [283,287,288,289]. Chamomile is generally regarded as safe when used appropriately; however, allergic reactions may occur, especially in individuals sensitive to other *Asteraceae* species [285]. The following sections will detail the specific chemical components of German chamomile and their scientifically supported activities, with a particular focus on their contribution to gut health.

German chamomile is renowned for its rich and varied chemical profile, which underpins its therapeutic actions. The most significant components are found in the essential oil and the flavonoid fraction of the flower heads. The essential oil, responsible for the plant’s characteristic aroma, contains a variety of terpenoids, with α-bisabolol, its oxides, and the blue sesquiterpene chamazulene being the most prominent. These compounds are highly valued for their potent anti-inflammatory and antiseptic properties [290,291,292,293]. The non-volatile fraction of the plant is dominated by flavonoids, especially apigenin and its glycosides, such as apigenin-7-O-glucoside. Other important flavonoids include luteolin, quercetin, and patuletin, which collectively contribute to the plant’s antioxidant and anti-inflammatory effects [283,287,288]. Additionally, the plant contains coumarins like umbelliferone and herniarin, which also possess anti-spasmodic properties [294,295]. The diverse phytochemical composition of German chamomile is of particular interest in the context of gut health. The plant’s polysaccharides and other non-digestible carbohydrates can act as a substrate for beneficial gut bacteria, potentially serving as a prebiotic agent [296,297]. Moreover, the antimicrobial properties of its essential oil components, such as α-bisabolol and chamazulene, may selectively inhibit the growth of pathogenic microbes, thereby contributing to a balanced gut ecosystem [298,299,300].

Beyond its calming aroma, German chamomile exhibits potent antioxidant and anti-inflammatory activities, which are fundamental to its gut-healing properties. The plant’s power lies in the synergistic action of its flavonoids, particularly apigenin and quercetin, and essential oil compounds [301,302]. These molecules function as powerful radical scavengers, neutralising ROS and guarding the intestinal lining against oxidative damage. This protective shield is crucial for maintaining the gut barrier’s integrity and preventing conditions like “leaky gut”, which can be a gateway to broader inflammatory issues [298,303,304,305]. Chamomile also enhance the body’s own antioxidant defences, increasing the expression of several antioxidant enzymes, such as heme oxygenase-1 (HO-1), peroxiredoxin-1 (Prx-1), and thioredoxin-1 (Trx-1) in a dose-dependent manner. This upregulation is mediated through the Keap1-Nrf2 signalling pathway, which enhances the nuclear translocation of Nrf2 and its binding to the antioxidant response element in the nucleus [306]. Additionally, chamomile induces the expression of NAD(P)H:quinone oxidoreductase, superoxide dismutase, and catalase, further supporting its role in enhancing antioxidant defences [307].

The anti-inflammatory effects of chamomile are equally significant. Its key components, apigenin and α-bisabolol, act at a molecular level to calm the inflammatory cascade. Apigenin inhibits the expression of major pro-inflammatory cytokines like TNF-α and IL-6, which are central to chronic gut inflammation. Moreover, apigenin’s anti-inflammatory effects are partly due to its ability to inhibit the activation of NF-κB, a key transcription factor involved in the inflammatory response. This inhibition reduces the production of inflammatory mediators [308,309]. Meanwhile, α-bisabolol and chamazulene work by inhibiting enzymes such as COX-2 and the production of prostaglandin E2 (PGE2), which are involved in the synthesis of pro-inflammatory mediators [310,311,312,313,314,315,316]. α-bisabolol reduces the production of nitric oxide (NO) and the expression of iNOS in various models, contributing to its anti-inflammatory effects. The anti-inflammatory effects of α-bisabolol are mediated through the inhibition of NF-κB and AP-1 signalling pathways, which are crucial for the expression of inflammatory genes [310,311,312,313]. Additionally, it affects the phosphorylation of extracellular signal-regulated kinase (ERK) and p38 mitogen-activated protein kinase (p38), but not c-Jun N-terminal kinase (JNK) [310]. α-bisabolol and chamazulene also exhibit other anti-inflammatory actions, such as reducing the production of pro-inflammatory cytokines like TNF-α and IL-6 [311,313,316]. This comprehensive approach effectively soothes intestinal inflammation, creating a more hospitable environment for a diverse and healthy gut microbiome.

One of chamomile’s most celebrated applications in digestive health is its ability to directly address common complaints like cramping and bloating. This is thanks to its spasmolytic (or muscle-relaxing) properties, primarily driven by the flavonoid apigenin and the coumarin herniarin [304,317,318,319]. These compounds soothe the smooth muscles of the intestinal walls, a crucial mechanism for relieving spasms and pain associated with conditions like irritable bowel syndrome. By calming these involuntary contractions, chamomile promotes smoother intestinal motility and provides rapid relief from discomfort [320,321].

Furthermore, chamomile’s protective benefits extend to reinforcing the physical structure of the gut. Chronic inflammation and oxidative stress can weaken the tight junctions that hold intestinal cells together, leading to increased permeability. By mitigating these two damaging factors, the flavonoids and terpenoids in chamomile help to preserve and restore these critical junctions [318,322,323,324]. Strengthening the intestinal barrier prevents the entry of toxins, pathogens, and other irritants into the bloodstream, a key step in preventing systemic inflammation and supporting overall gut health. This dual action of calming muscle spasms and reinforcing the gut barrier highlights chamomile’s comprehensive and powerful role in supporting a resilient digestive system [317,325,326].

Antibacterial and antifungal properties of chamomile are attributed to its essential oil content. The oil is characterised by a complex phytochemical profile, including sesquiterpenes (such as α-bisabolol, bisabololoxides A and B, and farnesene), sesquiterpene lactones (notably chamazulene, which imparts a distinctive blue coloration), and acetylene derivatives [327]. In the literature, extracts from various parts of the chamomile plant (flowers, leaves, and aerial parts) have been investigated, using aqueous [328,329] ethanolic [330,331,332,333,334], and methanolic [330], petroleum ether [330], chloroform [335], cyclohexane, ethyl acetate, hexane, diethyl ether solutions as extractants. It is hypothesised that the antimicrobial mechanism of chamomile extracts involves an effect on the cell wall, which explains their generally observed higher efficacy against Gram-positive bacteria [284]. Chamomile essential oils, due to their lipophilic nature, easily penetrate the cell walls and membranes of microorganisms. This leads to a disruption of their integrity, impairing the function of enzymatic systems and metabolic pathways essential for the survival of microorganisms, ultimately resulting in cell death [336]. Studies on chamomile oil nanoemulsions show that extracts increase superoxide and peroxide concentrations within bacterial cells (*E. coli*, *S. aureus*, etc.), inducing oxidative stress and bacterial death [337]. The analysed literature indicates that *Staphylococcus aureus* is the most sensitive to the effects of chamomile extracts, while *Pseudomonas aeruginosa* exhibits the highest resistance. Roby et al. [338] reported that among the essential oil (EO) of *M. chamomilla* and four different extracts, the EO exhibited the strongest antibacterial activity against all bacterial strains tested. In addition, the antibacterial effects of both the EO and the extracts increased in a dose-dependent manner. When analysing the antimicrobial effectiveness of various extracts, it appears that ethanol and methanol extracts are the most effective against *S. aureus* and *Pseudomonas aeruginosa*. In contrast, ethyl acetate extracts demonstrate greater efficacy in inhibiting the growth of *H. pylori*. However, the data in the literature are not entirely consistent, as the results often depend on the bacterial strain, the type of extract, or the geographical origin of the plant material [284].

Chamomile contains a rich profile of polyphenols, particularly derivatives of apigenin, luteolin, and quercetin [339]. Polyphenols are not classified as prebiotics; however, their characteristics are similar to those of classical oligosaccharides [340]. These compounds are not fully absorbed in the small intestine, allowing them to reach the colon [341] and serve as substrates for fermentation by beneficial gut bacteria, especially *Bifidobacterium* and *Lactobacillus* species. Their metabolism by gut microbes leads to the production of short-chain fatty acids (SCFAs) such as butyrate, propionate, and acetate, which strengthen the intestinal barrier and regulate immunity [341].

Chamomile (genus *Matricaria* or *Chamaemelum*) is widely recognised and extensively utilised in traditional medicine for its therapeutic properties, particularly in the management of gastrointestinal discomforts, spasms, inflammatory conditions, and other minor health disorders [327]. Chamomile extracts are used most frequently for oral cavity inflammatory conditions, even in infants (they are a common ingredient in toothing gels), as well as in the treatment of stomach inflammations.

Iberogast (STW 5) is a well-studied herbal preparation that has been tested in numerous clinical trials. It is a herbal medicine containing extracts from nine plants, including chamomile flowers. The anti-inflammatory effects of STW 5 are broad, involving the inhibition of inflammatory mediators and antioxidant activity, which may play an important role in alleviating symptoms of IBD. Experimental studies have shown that STW 5 prevents increased intestinal permeability in the proximal colon, jejunum, and distal intestine. Moreover, it has been demonstrated that STW 5 induces significant changes in the composition of gut microbiota, contributing to its normalisation by, for instance, reducing the abundance of bacteria belonging to the genera *Clostridium* and *Bacteroidetes* [342,343].

### 4.4. Pot Marigold (Calendula officinalis L.)

**Figure 6:**
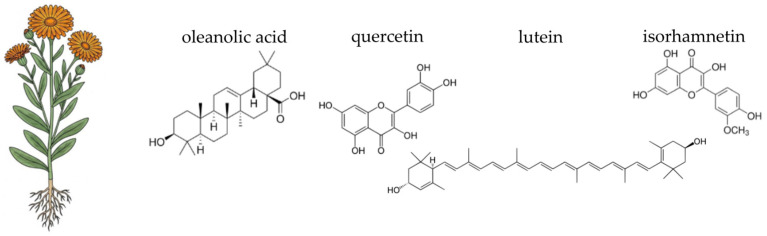
Botanical illustration of pot marigold and the chemical structures of selected four key bioactive compounds characteristic for this plant.

Pot marigold (*Calendula officinalis* L.), a flowering plant of the *Asteraceae* family, is widely cultivated in temperate regions and has a long history of use in both traditional medicine and modern pharmacology (Figure 6). It is frequently used in pharmaceutical and dermatological products intended for the care of irritated or damaged skin. Topical calendula preparations, such as ointments, creams, and gels, are commonly applied to accelerate wound healing, reduce inflammation in minor burns, and manage skin conditions such as nappy rash, eczema, or radiotherapy-induced dermatitis [344,345,346,347]. Oral and oromucosal formulations, including mouthwashes and lozenges, are also used to alleviate inflammation in cases of gingivitis, sore throat, or oral mucositis [348,349,350]. Additionally, calendula extracts are included in gastrointestinal products aimed at relieving gastritis, mucosal irritation, and mild cramping [344,351,352]. *Calendula officinalis* is generally considered safe for topical and internal use when appropriately dosed; however, allergic reactions may occur in individuals with hypersensitivity to other members of the *Asteraceae* family [344]. The following sections will detail the specific chemical components of pot marigold and their scientifically supported activities, with a particular focus on their contribution to gut health.

The therapeutic efficacy of pot marigold (*Calendula officinalis*) is underpinned by its rich and diverse phytochemical profile, which is particularly concentrated in the flower heads [353]. The most significant components are found in the essential oil, the flavonoid, and the terpenoid fractions [354,355,356,357]. The essential oils from both the leaves and flowers are characterised by a high concentration of α-cadinol, a prominent sesquiterpene alcohol, which constitutes over 30% of the total essential oil content [355,356]. Pot marigold seed oil is notable for its unique fatty acid composition, particularly the high content of conjugated linolenic acids, including α-calendic acid [358,359]. The plant’s non-volatile fraction is dominated by flavonoids and phenolic compounds. Key flavonoids identified include rutin, quercetin-3-O-glucoside, and isorhamnetin-3-O-glucoside. Furthermore, *C. officinalis* contains a variety of triterpenoids, such as oleanolic acid derivatives and triterpenoid saponins (including calendulosides) [360,361]. The vibrant orange and yellow colours n of the petals are due to high concentrations of carotenoids, such as lutein, lycopene, and β-carotene [362,363]. Moreover, pot marigold includes polysaccharides, particularly acidic pectic type polysaccharides, which can be extracted from industrial waste products of the plant. These polysaccharides have specific chemical properties and potential applications in various industries, including drug delivery systems, food additives, and possibly in cosmetic formulations [64,364].

*Calendula officinalis*, commonly known as pot marigold, contains several compounds responsible for its antioxidant activity, each with distinct mechanisms of action. The primary compounds identified include flavonoids, carotenoids, phenolic acids, triterpenoids, and essential oils. Flavonoids, like rutin, quercetin-3-O-glucoside, and isorhamnetin-3-O-glucoside, are known for their ability to scavenge free radicals and reduce oxidative stress through hydrogen donation and one-electron reduction mechanisms [360,365]. The carotenoids, such as β-carotene, work as potent radical scavengers, neutralising ROS and protecting the intestinal mucosa from oxidative damage [353,354]. Chlorogenic acid and 3,4-dicaffeoylquinic acid are significant phenolic acids found in *C. officinalis*. These compounds contribute to antioxidant activity by neutralising free radicals and reducing lipid peroxidation [360,366]. They also play a role in modulating gene expression related to apoptosis and cell cycle progression, enhancing cytotoxicity against cancer cells [367]. C16-hydroxylated triterpenoids are key contributors to the anti-inflammatory and antioxidant activities of *C. officinalis*. They modulate interleukin 6 release, which is crucial in inflammatory responses [368]. Triterpenoids also exhibit neuroprotective effects by attenuating oxidative stress and neuronal damage [369,370]. The essential oils from *C. officinalis*, particularly sesquiterpene alcohols like α-cadinol, demonstrate strong antioxidant capacity through free radical scavenging and reducing mechanisms. These oils also inhibit enzymes such as amylase, glucosidase, acetylcholinesterase, and butyrylcholinesterase, contributing to their therapeutic potential [356]. Additional compounds such as gallic acid, scopoletin-7-O-glucoside, and calenduloside E have been identified for their antioxidant properties. These compounds enhance the plant’s ability to protect against oxidative stress and inflammation [360,366].

*Calendula officinalis* exhibits significant anti-inflammatory properties attributed to various bioactive compounds. For instance, C16-hydroxylated triterpenoids modulate the release of interleukin 6, a cytokine involved in inflammation, thereby contributing to the anti-inflammatory activity of *Calendula officinalis* [368]. Found in *Calendula officinalis* tincture, flavonol glycosides stimulate fibroblast proliferation and migration through a phosphoinositide 3-kinase (PI3K)-dependent pathway, enhancing wound healing and reducing inflammation [371]. Triterpene esters (faradiol-3-myristate and its aglycone) inhibit NF-κB driven transcription, which is a key pathway in the inflammatory response, particularly in gastric inflammation [372]. Isolated from the roots of *Calendula officinalis*, prenylated acetophenones decrease lipopolysaccharide-stimulated NO production in J774.1 cells, thus reducing inflammation [373]. Phenolic acids, flavonols, and coumarin modulate the expression of genes involved in apoptosis and cell cycle progression, such as BCL2, BAX, NF-κB, and STAT3, contributing to anti-inflammatory and cytotoxic effects against cancer cells [367]. The volatile compounds, like α-thujone, β-thujone, 4-terpineol, eucalyptol, α-cadinol, and α-epi-muurolol, exhibit anti-inflammatory properties through various mechanisms, including antimicrobial and antifungal activities [374]. Chlorogenic acid, 3,4-dicaffeoylquinic acid, rutin, isorhamnetin 3-O-glucoside, and calenduloside E activate the PI3K/Akt signalling pathway and inhibit the ERK signalling pathway, preventing neuronal death and alleviating inflammation-related neuronal degeneration [366].

In addition to its antioxidant and anti-inflammatory actions, pot marigold is particularly valued for its ability to promote mucosal healing and protect the integrity of the intestinal barrier. This effect is primarily attributed to its high concentration of polysaccharides and triterpenoids. Polysaccharides have been shown to stimulate cell proliferation and tissue regeneration, which is crucial for repairing damaged intestinal linings. This is analogous to its well-documented effects on wound healing in the skin, where it promotes the formation of new tissue and enhances re-epithelialisation [375,376,377,378].

The triterpenoids also play a crucial role by providing cytoprotective effects, shielding intestinal cells from damage. This, combined with the plant’s anti-inflammatory properties, helps to preserve and restore the function of the tight junctions between intestinal epithelial cells. A strong intestinal barrier is vital for preventing the translocation of toxins, undigested food particles, and pathogens into the bloodstream, which can trigger systemic inflammation and immune responses. The ability of *Calendula officinalis* to both heal existing mucosal damage and strengthen the gut barrier underscores its comprehensive role in supporting overall digestive health and function [379].

In Karnwal’s study [380], the antimicrobial effects of aqueous, ethanol, and methanol extracts of marigold were tested against seven food-poisoning causing bacterial spp. Methanol extracts showed the highest efficacy against *Staphylococcus aureus* and *Pseudomonas aeruginosa*. Unfortunately, the cited paper did not characterise the individual extracts based on their composition. The author, referencing the work of [381], explains the high antimicrobial effectiveness by the presence of catechins, whose molecules act directly on the cell wall of pathogenic bacteria, leading to cell death. Ethanol extracts of *C. offcinalis* were used against *C. pylori* by Cwikla et al. [382] showed antibacterial properties. In Darekar et al. [383] antibacterial effects of different extracts of *C. officinalis* flowers against Gram-negative and Gram-positive bacteria were determined by disc diffusion method. From all tested extracts (chloroform, ethanol, methanol, water, n-hexane, ethyl acetate, toluene, DMSO) chloroform extracts showed the most efficient antibacterial effects against tested strains of Gram-positive (*Staphylococcus aureus*, *Bacillus subtilis* and *Enterococcus faecalis*) and one strain of Gram-negative (*Klebsiella pneumonia*) bacteria [383]. Among the tested organisms, *Escherichia coli* and *Klebsiella pneumoniae* were the most sensitive, showing the largest zones of inhibition. In contrast, *Bacillus subtilis* and *Sarcina lutea* demonstrated greater resistance to the extracts. Notably, the petroleum ether and chloroform extracts of *C. officinalis* displayed significant antibacterial activity, particularly against *E. coli* and *B. subtilis* [384]. *Calendula officinalis* leaf extracts exhibited varying degrees of antimicrobial activity against different microbial strains. Bioactive compounds responsible for antibacterial activity are: flavonoids (quercetin, isorhamnetin, rutin)—key phenolic compounds contributing to antioxidant and antimicrobial properties—triterpenoids, and essential oil constituents—enriched in sesquiterpenes (e.g., α-cadinene, γ-cadinene), which cause disruption of microbial membranes [346,368,385].

Dietary fibre content in flowers of *C. officinalis* is 62.33 g·100 g^−1^ and it is the highest fibre content compared to investigated edible flowers [386].

In traditional medicine, *C. officinalis* extracts are used for wound healing. Scientific studies indicate that calendula possesses anti-inflammatory, wound-healing, and analgesic properties, which contribute to the acceleration of mucosal healing and reduction of pain associated with aphthous ulcers. Its anti-inflammatory activity may also alleviate gingival inflammation by decreasing erythema, oedema, and bleeding. Furthermore, calendula-based mouth rinses have demonstrated potential in relieving pharyngeal discomfort and supporting the management of oropharyngeal infections. In post-operative dental settings, calendula may facilitate tissue regeneration and lower the risk of secondary infections [348]. In vitro investigations of calendula alcohol extract have demonstrated beneficial biological effects, such as stimulating the growth and movement of human fibroblasts and keratinocytes, fostering angiogenesis, and inhibiting collagenase [387]. A meta-analysis by Givol et al. [387] indicates that there is therapeutic potential in calendula extracts for treating wounds and burns. However, there is a lack of large clinical trials with established methodology. The trials conducted so far differ methodologically, making it impossible to draw clear conclusions on the efficacy of *C. officinalis* extracts [387]. Promising results are observed in the studies showing influence of *C. officinalis* on vaginosis [388]. In a study, Tedeschi et al. [389] found that vaginal gel comprising isoflavones, *Lactobacillus sporogenes*, and *C. officinalis* could significantly reduce the signs and symptoms of vaginal dystrophy (itching, burning, vulvovaginal erythema, and vaginal dryness) compared to placebo in postmenopausal women [389].

No clinical trials relating to the gastrointestinal tract have been conducted in the past five years.

### 4.5. Ceylon Cinnamon (Cinnamomum verum J. Presl)

**Figure 7:**
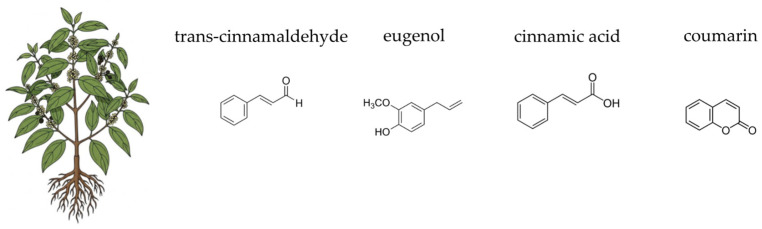
Botanical illustration of Ceylon cinnamon and the chemical structures of selected four key bioactive compounds characteristic for this plant.

Ceylon cinnamon (*Cinnamomum verum* J. Presl), commonly known as “true cinnamon,” is an evergreen tree belonging to the *Lauraceae* family (Figure 7). Native to Sri Lanka and southern India, it has a long-standing history of culinary and medicinal use [390,391]. In human applications, Ceylon cinnamon has been investigated for its supportive role in glycaemic control, lipid regulation, and gastrointestinal comfort. Clinical studies have shown its potential in reducing postprandial blood glucose levels, improving insulin sensitivity, and alleviating mild dyspeptic symptoms [392,393,394,395]. Traditionally used as a culinary spice or herbal infusion, Ceylon cinnamon is now widely available as a dietary supplement in standardised extract form, particularly in capsules and powders designed for metabolic support [396,397]. Due to its milder flavour and substantially lower coumarin content compared to *Cinnamomum cassia*, it is generally regarded as a safer option for long-term consumption. The plant is considered safe when used within established dietary limits, though allergic reactions may occur in sensitive individuals [398,399,400]. The following sections will detail the specific chemical components of Ceylon cinnamon and their scientifically supported activities, with a particular focus on their contribution to gut health.

The therapeutic properties of Ceylon cinnamon are attributed to a complex mixture of bioactive compounds found in its bark and leaves. The most prominent component of its essential oil is cinnamaldehyde, which is responsible for the plant’s characteristic aroma and much of its antimicrobial activity [401,402]. Other important volatile compounds include eugenol and linalool [402,403]. The plant is also rich in non-volatile compounds, particularly polyphenols, including flavonoids such as catechins, epicatechins, and procyanidins (specifically A-type procyanidins), which are powerful antioxidants [404]. The bark also contains a significant amount of dietary fibre, including mucilages and tannins, which contribute to its gut-supportive effects [404]. The unique chemical profile of *Cinnamomum verum*, particularly its low content of the hepatotoxic compound coumarin (a key differentiator from *Cinnamomum cassia*), makes it a preferred choice for therapeutic applications, especially for long-term use [405]. This blend of essential oils, polyphenols, and fibre acts synergistically to provide a multifaceted approach to improving digestive health and modulating the gut environment [406,407,408,409].

The antioxidant activity of *Cinnamomum verum* is attributed to several key compounds, each with specific mechanisms of action. Cinnamaldehyde is a major constituent of the essential oil of *Cinnamomum verum* and exhibits significant antioxidant activity. It acts by scavenging free radicals such as DPPH and ABTS radicals, and also shows metal chelating activity. Additionally, cinnamaldehyde inhibits lipid peroxidation, which helps in protecting cell membranes from oxidative damage [410,411,412,413]. Found predominantly in the leaf oil, eugenol is another potent antioxidant. It scavenges free radicals, particularly DPPH radicals, and exhibits strong reducing power. Eugenol also contributes to the inhibition of lipid peroxidation and has been shown to have metal chelating properties, enhancing its antioxidant efficacy [412,413,414]. Cinnamic acid and its derivatives are known for their antioxidant properties. Cinnamic acid works by scavenging free radicals and enhancing the antioxidant defence system. It also shows vasodilatory effects, which can improve blood flow and reduce oxidative stress in tissues [415,416]. *Cinnamomum verum* contains various phenolic compounds such as quercetin, gallic acid, rutin, and kaempferol. These compounds are effective free radical scavengers and contribute to the overall antioxidant activity by reducing oxidative stress and inhibiting lipid peroxidation [414,415]. Proanthocyanidins, a class of polyphenols found in cinnamon, exhibit strong antioxidant activity. They work by neutralising free radicals and protecting cells from oxidative damage. Proanthocyanidins also enhance the body’s antioxidant defence mechanisms [415]. Compounds like quercetin and kaempferol, found in cinnamon, are known for their antioxidant properties. They scavenge free radicals, inhibit lipid peroxidation, and enhance the activity of antioxidant enzymes such as SOD and glutathione peroxidase (GSH-Px) [414,415]. Cinnamyl acetate and cinnamic alcohol also contribute to the antioxidant activity of *Cinnamomum verum* by scavenging free radicals and inhibiting oxidative processes [406,415]. Studies have shown that cinnamon extracts can maintain high transepithelial electrical resistance and decrease tight junction permeability in intestinal cells, which are indicators of a healthy gut barrier. This is essential for preventing the entry of harmful substances and maintaining overall gut health [417].

The anti-inflammatory activity of *Cinnamomum verum* is attributed to several key compounds. Trans-cinnamaldehyde (TC) significantly reduces lipopolysaccharide (LPS)-dependent interleukin 8 secretion in THP-1 monocytes. It also mitigates the phosphorylation of protein kinase B (Akt) and nuclear factor NF-κB inhibitor alpha (IκBα), which are crucial in the inflammatory signalling pathways [418]. Additionally, TC interacts with toll-like receptors (TLR2 and TLR4), reducing inflammation [418,419]. Similar to trans-cinnamaldehyde, p-cymene reduces LPS-dependent IL-8 secretion and mitigates the phosphorylation of Akt and IκBα, contributing to its anti-inflammatory effects. Cinnamic acid, when combined with TC, shows synergistic anti-inflammatory effects by reducing IL-8 secretion and influencing the same signalling pathways [418]. Found in the essential oil of *Cinnamomum verum*, eugenol exhibits significant anti-inflammatory activity by reducing the production of nitric oxide and pro-inflammatory cytokines such as tumour necrosis factor-alpha and interleukin-1 beta (IL-1β) [406]. Cinnamyl alcohol also shows synergistic effects with trans-cinnamaldehyde, enhancing the overall anti-inflammatory activity [418]. Present in *Cinnamomum verum*, coumarin significantly attenuates the expression of inflammatory mediators like IL-1β, IL-8, and COX-2 by downregulating the NOX2/ROS and PKCδ/JNK/AP-1/NF-κB signalling pathways [420]. Proanthocyanidins exhibit anti-inflammatory properties by targeting multiple metabolic pathways involved in inflammation, including the inhibition of NF-κB nuclear translocation [415]. Cinnamate contributes to the anti-inflammatory effects by modulating the Toll-like receptor signalling pathway, which is crucial in the inflammatory response [419].

Cinnamon is among the most extensively studied medicinal plants due to its potent antimicrobial activity. Its therapeutic use against microbial infections has a long history, rooted in traditional medicine. The antimicrobial potential of cinnamon is primarily attributed to its diverse array of bioactive compounds, including cinnamaldehyde, cinnamate, cinnamic acid, and numerous essential oils such as trans-cinnamaldehyde, cinnamyl acetate, eugenol, L-borneol, camphor, caryophyllene oxide, β-caryophyllene, L-bornyl acetate, E-nerolidol, α-cubebene, α-terpineol, terpinolene, and α-thujene [421]. These secondary metabolites exhibit broad-spectrum antimicrobial properties [422]. Cinnamaldehyde, a bioactive component of cinnamon, was shown to exhibit more potent in vitro antibacterial properties against 5 common foodborne pathogenic bacteria (*Bacilluscereus*, *Listeria monocytogenes*, *S. aureus*, *E. coli*, and *Salmonella*), with MIC being 125 to 500 µg·mL^−1^ as compared to crude cinnamon stick extract (625 to >2500 µg·mL^−1^) [423]. Trans-cinnamaldehyde is the most thoroughly characterised compound in this context. Its mechanism of action involves disruption of the bacterial cell membrane due to its ability to penetrate the lipid bilayer and bind membrane-associated proteins, impairing cell wall synthesis [424]. This leads to loss of membrane integrity, reduced protein functionality, and eventual denaturation of intracellular enzymes. Gram-positive bacteria are particularly susceptible to TC [425], whereas Gram-negative bacteria such as *Escherichia coli* require higher concentrations for effective inhibition [426]. In addition to membrane disruption, TC also inhibits ATP synthesis by downregulating F1F0-ATPase expression, as demonstrated in *Cronobacter sakazakii* [427,428]. At higher concentrations, TC acts as a direct ATPase inhibitor [429]. Moreover, TC interferes with bacterial cell division. Confocal microscopy studies have shown that TC disrupts the spatial arrangement of the Z-ring in *E. coli*, reducing its formation frequency by nearly 50%. This effect is mediated by inhibition of GTP-dependent FtsZ polymerisation, interference with Z-ring assembly, and binding to specific regions of FtsZ, as observed in *Bacillus cereus* [430,431]. These multifaceted antimicrobial mechanisms highlight the potential of cinnamon-derived compounds, particularly trans-cinnamaldehyde, as natural antimicrobial agents and promising candidates for further pharmaceutical development. In addition to its membrane-disrupting and cell division-inhibiting properties, TC also impairs bacterial motility and inhibits biofilm formation. Studies on *Enterobacter sakazakii* demonstrated that TC downregulated genes associated with the flagellar apparatus (e.g., fliD, flgJ, motA, motB), leading to a significant reduction in motility and, consequently, biofilm formation [427]. The antibiofilm effects of TC have been observed across a wide range of clinically relevant pathogens, including methicillin-resistant *Staphylococcus aureus* and *Staphylococcus epidermidis* [432,433,434], *Burkholderia* spp. [435], *E. coli*, *Pseudomonas* spp. [436], *Listeria* spp. [437], *Salmonella* spp. [434,438], and *Vibrio* spp. [439]. Biofilm formation is closely associated with quorum sensing (QS) mechanisms, which TC is also known to disrupt. It has been shown to downregulate QS-related genes such as *bcsA* and *luxR*, and to interfere with autoinducer signalling molecules without affecting bacterial growth in *Vibrio* spp. [440]. TC also inhibited QS activity in *E. coli*, *Pseudomonas aeruginosa*, and *S. pyogenes*, further supporting its potential as an anti-virulence agent [436]. Several studies have reported that trans-cinnamaldehyde exhibits limited stability upon exposure to air, due to its reactive unsaturated aldehyde group, which is readily oxidised to cinnamic acid. This oxidative conversion contributes to its volatility and chemical instability [441]. Furthermore, in vivo, TC may undergo enzymatic degradation before exerting its bactericidal effects. Once absorbed into the bloodstream, TC is rapidly and irreversibly metabolised to cinnamic acid, thereby limiting its bioavailability and therapeutic potential [442].

Cinnamon extract demonstrated a moderate stimulatory effect on the growth of *Lactobacillus* strains. At a concentration of 1.13 mg·mL^−1^, growth was enhanced in 5 strains, and at 2.25 mg·mL^−1^, 7 strains showed increased growth [443]. In the study of [444] showed that cinnamon also supported the growth of probiotic strains such as *Lactobacillus reuteri* and *L. rhamnosus*, particularly at 1.13 and 2.25 mg·mL^−1^. While its effect on *Bifidobacterium* spp. was modest compared to other spice extracts, its ability to promote *Lactobacillus* growth indicates potential prebiotic-like activity that may contribute to gut health. In vivo study showed that cinnamaldehyde did not modulate the population of selected *Lactobacillus* and *Bifidobacterium* counts in mouse faecal content [445].

Current in vitro and animal in vivo studies indicate that cinnamon exhibits a broad spectrum of biological activities, including anti-inflammatory, antimicrobial, antioxidant, antitumor, cardioprotective, hypocholesterolemic, and immunomodulatory effects. Evidence from in vitro experiments suggests that cinnamon may function as an insulin mimetic, enhance insulin signalling, or stimulate cellular glucose metabolism. Likewise, animal models have consistently demonstrated pronounced hypoglycaemic effects. Nevertheless, the limited number of well-controlled clinical trials constrains the ability to draw definitive conclusions regarding the potential health benefits of cinnamon in humans. Among the various applications, the adjunctive use of cinnamon in the management of type 2 diabetes mellitus appears most promising; however, further high-quality clinical research is essential before firm therapeutic recommendations can be established [446,447].

A randomised controlled trial by Park et al. [448] evaluated the effects of cinnamon water extract (CWE) in 70 subjects with diarrhoea over an 8-week period. Participants received either 400 mg CWE capsules or placebo twice daily. CWE significantly increased colonic transit time and beneficial faecal metabolites while reducing harmful compounds. It also improved gut microbiota diversity, notably increasing *Bifidobacterium longum* levels, which correlated with improved stool consistency. Overall, CWE alleviated diarrhoea symptoms through microbiota-related metabolic changes [448].

Separately, numerous clinical trials suggest that cinnamon supplementation, particularly in capsule form and at doses of ≤2 g per day, improves glycemic and lipid profiles and reduces BMI, especially in patients with type 2 diabetes (T2DM) [449,450].

### 4.6. Dandelion (Taraxacum officinale F.H. Wigg.)

**Figure 8:**
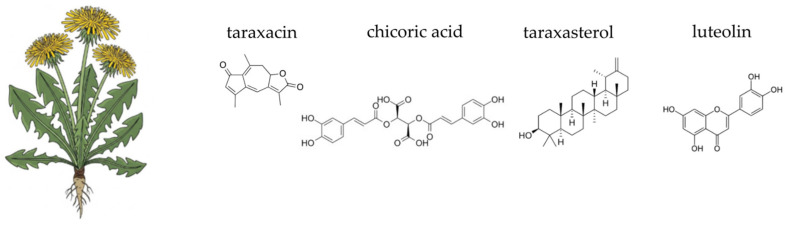
Botanical illustration of dandelion and the chemical structures of selected four key bioactive compounds characteristic for this plant.

Dandelion (*Taraxacum officinale* F.H. Wigg.), a well-known perennial herb from the *Asteraceae* family, is widely distributed across temperate regions of the Northern Hemisphere (Figure 8) [451,452]. Traditionally, all parts of the plant—from roots to flowers—have been used in various medicinal systems for their diuretic, hepatoprotective, and digestive-supportive properties. In modern phytotherapy, dandelion is primarily valued for its role in promoting liver and gallbladder health, stimulating appetite, and as a mild laxative. Its therapeutic efficacy is attributed to a rich array of bioactive compounds, including sesquiterpene lactones, phenolic acids, flavonoids, and fructans [453,454,455,456]. Dandelion is generally considered safe for consumption; however, individuals allergic to plants in the *Asteraceae* family (such as ragweed or chrysanthemums) should use it with caution, and its diuretic properties may interact with certain medications [457,458]. The following sections will detail the specific chemical components of dandelion and their scientifically supported activities, with a particular focus on their contribution to gut health.

The therapeutic benefits of dandelion are derived from a diverse phytochemical profile, with significant variations between the roots and leaves. The roots are particularly rich in sesquiterpene lactones, such as taraxacin and taraxinic acid, which are responsible for the plant’s bitter taste, liver tonic effects and its ability to stimulate appetite and bile flow [459,460]. The roots are also a primary source of fructans, predominantly inulin and fructooligosaccharides, which constitute a significant portion of its dry weight [454,460]. These fructans have prebiotic effects, promoting gut health and aiding in digestion [460,461]. The leaves are rich in flavonoids, including luteolin and quercetin, as well as phenolic acids, such as chicoric acid and chlorogenic acid [461,462,463]. These compounds are particularly effective in mitigating oxidative stress and inflammation [462]. The leaves also contain significant amounts of potassium, which helps counterbalance the diuretic effects of other components, making it a “potassium-sparing” diuretic in some contexts [460,464,465]. The synergistic interaction of these various classes of compounds provides a comprehensive approach to promoting digestive and metabolic health.

*Taraxacum officinale* exhibits significant antioxidant activity attributed to various bioactive compounds and their mechanisms of action. The primary compounds responsible for this activity include phenolic compounds, flavonoids, and specific acids such as caffeic acid, chlorogenic acid, and cichoric acid. Phenolic compounds and flavonoids are abundant in *T. officinale* and contribute to its antioxidant properties by scavenging reactive oxygen species and reactive nitrogen species. The phenolic compounds, including caffeic acid and chlorogenic acid, are particularly effective in neutralising free radicals such as DPPH, NO, OH, and H_2_O_2_, thereby protecting cells from oxidative damage [466,467]. Identified as a major polyphenolic compound in *T. officinale* tincture, cichoric acid plays a crucial role in reducing oxidative stress markers such as total oxidative stress (TOS), oxidative stress index (OSI), and malondialdehyde (MDA). It also enhances the total antioxidant capacity (TAC) and reduces nitrites/nitrates (NOx) levels, contributing to its antioxidant and anti-inflammatory effects [467,468]. The triterpenoid taraxasterol exhibits strong antioxidant activity by inhibiting oxidative stress, inflammation, and apoptosis. It achieves this by promoting the expression of Nrf2 and heme oxygenase-1 (HO-1), while suppressing JNK phosphorylation and reducing the Bax/Bcl-2 ratio and caspase-3 expression. These actions collectively protect cells from oxidative damage and apoptosis [469,470,471]. Found in *T. officinale* extracts, ursolic acid contributes to its antioxidant activity by inhibiting the proliferation of cancer cells and protecting normal cells from oxidative stress. It works synergistically with other acids like caffeic and chlorogenic acids to enhance the overall antioxidant effect [471]. Dandelion’s beneficial effects on the gut are driven by a powerful synergy of its bioactive compounds.

*Taraxacum officinale* exhibits significant anti-inflammatory activity due to various bioactive compounds and their mechanisms of action. The pentacyclic triterpene, taraxasterol, inhibits LPS-induced production of pro-inflammatory cytokines such as TNF-α and IL-1β, and suppresses NF-κB activation. It also disrupts the formation of lipid rafts and inhibits the translocation of TLR4 into lipid rafts, thereby reducing inflammation. Additionally, taraxasterol activates the liver X receptor alpha-ATP-binding cassette transporter A1 (LXRα-ABCA1) signalling pathway, which induces cholesterol efflux from cells, further contributing to its anti-inflammatory effects [472]. Taraxasterol acetate (TSA) has shown promising anti-inflammatory effects in vitro and in vivo, particularly in the context of ulcerative colitis (UC). TSA alleviates clinical symptoms, repairs the intestinal barrier, and restores gut microbiota structure and metabolic levels in UC models [473]. Polysaccharides (TOPs) reduce the expression of iNOS and TNF-α in LPS-stimulated cells. They inhibit the phosphorylation of NF-κB and its upstream signalling molecule PI3K/Akt, and induce heme oxygenase (HO)-1 via the nuclear translocation of Nrf2, providing both anti-inflammatory and antioxidative benefits [474]. Caffeic acid, chlorogenic acid, and ursolic acid exhibit anti-proliferative effects on cancer cell lines and have been shown to possess cytotoxic properties. Their mechanisms include the inhibition of cell viability and potential interactions with conventional chemotherapeutics [471]. Cichoric acid, identified in *Taraxacum officinale* tincture, contributes to the reduction of oxidative stress markers such as total oxidative stress, oxidative stress index, and total antioxidant capacity. It also decreases levels of malondialdehyde, thiols (SH), and NOx, and reduces the expression of inflammatory markers like NF-κB [467].

Furthermore, the bitter sesquiterpene lactones in dandelion roots provide a unique digestive benefit by stimulating the production and flow of bile from the liver and gallbladder [454]. This choleretic and cholagogue action improves the digestion and absorption of dietary fats and fat-soluble vitamins. By enhancing bile flow, dandelion helps alleviate common digestive symptoms like bloating and indigestion, contributing to a more efficient and comfortable digestive process [460]. This combination of nourishing the microbiome and optimising digestion makes dandelion a valuable addition to a gut-supportive regimen.

In traditional medicine, all anatomical parts of *Taraxacum officinale*, including the roots, leaves, stems, and flowers, have been utilised for antimicrobial applications. The disruption of the bacterial cell membrane is primarily attributed to sesquiterpenes present in the essential oil (e.g., α-cadinene, γ-cadinene). Damage to membrane integrity leads to the leakage of intracellular components. Another antimicrobial mechanism, such as the inhibition of bacterial enzymes, is associated with flavonoids like quercetin and isorhamnetin. Carotenoids such as β-carotene and lycopene may indirectly contribute to the antimicrobial properties of dandelion through their antioxidant activity. Additionally, certain phenolic compounds interfere with the quorum sensing process, which is essential for biofilm formation and the coordination of bacterial population behaviours. QS inhibition reduces the ability of bacteria to form biofilms and produce virulence factors. In the analysed literature, as with other medicinal plants, there is no scientific consensus regarding antimicrobial activity. The effectiveness depends on the specific plant part used, the type of extract, as well as the species or even the strain of the microorganism. According to Molan et al. [475], both ethanol and aqueous extracts of dandelion exhibit antimicrobial activity; however, ethanol extracts are more effective at lower concentrations, with *Escherichia coli* being the most susceptible to their effects. Diaz et al. [476] reported that dandelion extracts displayed pronounced antibacterial activity against multiple bacterial species, including *Bacillus subtilis*, *Staphylococcus aureus*, *Escherichia coli*, *Klebsiella pneumoniae*, and *Pseudomonas aeruginosa*. In the study by Ionescu et al. [477], the researchers investigated the antimicrobial properties of hydroalcoholic extracts from dandelion. Like previous findings, antimicrobial activity was observed against *E. coli* and *S. abony*, whereas no activity was detected against *S. aureus*.

Due to its polysaccharide content, dandelion is considered a prebiotic. In the study by Cao et al. [478], it was demonstrated that polysaccharides isolated through extraction from whole dried plants at a liquid-to-dry matter ratio of 25 mL·g^−1^ had the highest polysaccharide concentration. Furthermore, in vitro fermentation chamber experiments showed an increase in the abundance of microbiota belonging to the genera *Bifidobacterium*, *Olsenella*, and *Dialister* [478].

*Taraxacum officinale*, or dandelion, has been used in traditional medicine to treat liver, kidney, lung disorders, and diabetes.

Seventy women were randomised to receive either DAPAD (D-mannose, citric acid, prebiotic fibres, *Astragalus*, and dandelion) or placebo. The DAPAD group showed significantly higher clinical and bacteriological resolution at days 6 and 35 compared to placebo, with no moderate or severe symptoms at day 35. Three mild, unrelated adverse events were reported, indicating that DAPAD is an effective and safe treatment for acute uncomplicated *E. coli* urinary tract infections (UTIs) [479].

In a separate study, the effect of dandelion on reducing mastitis in breastfeeding women was investigated. It was found that dandelion granules increased microbial diversity in breast milk samples [480].

### 4.7. Fennel (Foeniculum vulgare Mill.)

**Figure 9:**
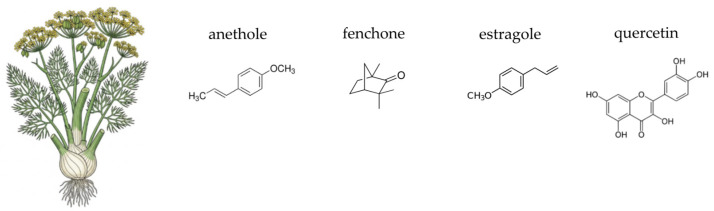
Botanical illustration of fennel and the chemical structures of selected four key bioactive compounds characteristic for this plant.

*Foeniculum vulgare* Mill., commonly known as fennel, is a perennial aromatic herb belonging to the *Apiaceae* family (Figure 9). Indigenous to the Mediterranean region, it has been extensively utilised since antiquity in both culinary and medicinal contexts. Historical records indicate its use in ancient Greek, Roman, Egyptian, and Indian traditions, particularly for its carminative, galactagogue, and anti-inflammatory properties, as well as for supporting respiratory and gastrointestinal health [481,482]. Traditionally, fennel was administered as a herbal infusion or decoction, or incorporated directly into the diet as both a vegetable and spice [483,484,485]. In contemporary phytotherapy, it is available in various forms, including standardised extracts, essential oils, and dried fruit preparations, widely used for ailments such as dyspepsia, infant colic, dysmenorrhoea, and mild respiratory complaints [482,486,487,488]. The edible parts of the plant—most notably the seeds, bulbs, and leaves—are consumed fresh, dried, or cooked, and are also used in the production of teas, tinctures, and essential oils [482,489,490]. *Foeniculum vulgare* is generally safe for short-term use, but prolonged use can lead to allergic reactions, atopic dermatitis, and photosensitivity. It is recommended to limit use to a maximum of two weeks to minimise side effects and interactions [491]. Fennel tincture and oils can be irritants to skin and eyes and act as dermal and respiratory sensitizers. Proper handling and minimising exposure are advised to reduce risks [492]. Moreover, fennel can also inhibit cytochrome P450 3A4 (CYP3A4), affecting the pharmacokinetics of drugs metabolised by this enzyme. Significant interactions with drugs like ciprofloxacin have been noted [491,493,494].

The therapeutic properties of fennel are largely attributed to its volatile essential oil, which is particularly concentrated in its fruits (commonly referred to as “seeds”). The primary bioactive compound is trans-anethole, an aromatic ether responsible for the plant’s characteristic anise-like flavour and a significant portion of its pharmacological effects [495,496]. Other key components of the essential oil include fenchone and estragole, which contribute to its anti-inflammatory and antimicrobial properties [495,497,498,499]. Fennel also contains a range of non-volatile compounds, including flavonoids like quercetin, rutin, and kaempferol, which are well-known for their potent antioxidant activity [485,500,501]. The plant is also a good source of dietary fibre, including mucilages, which provide bulk and act as a prebiotic [482,496,502]. The synergistic interaction of these various classes of compounds—from volatile essential oils to non-volatile flavonoids and fibre—provides a comprehensive approach to improving gastrointestinal health and modulating the gut environment.

The antioxidant activity of *Foeniculum vulgare* is attributed to several compounds, primarily phenolic acids, flavonoids, and essential oil components. These compounds exhibit their antioxidant effects through various mechanisms, including free radical scavenging, metal chelation, and inhibition of oxidative enzymes. Caffeic acid, quinic acid, and chlorogenic acid are major phenolic compounds found in fennel extracts. These compounds contribute significantly to the antioxidant activity by scavenging free radicals and chelating metal ions, which prevents the formation of reactive oxygen species [503]. Trans-anethole, limonene, fenchone, and estragole are prominent components of fennel essential oils. These compounds exhibit strong antioxidant activities by scavenging free radicals and inhibiting lipid peroxidation [504,505,506]. Trans-anethole, in particular, has been shown to inhibit oxidative enzymes and enhance the activity of endogenous antioxidant enzymes like superoxide dismutase and catalase, thereby reducing oxidative stress [507]. Compounds such as α-pinene, myrcene, and γ-terpinene also contribute to the antioxidant activity of fennel. These terpenes can neutralise free radicals and inhibit oxidative damage to biomolecules [504,505].

The anti-inflammatory effects of fennel are equally important. Trans-anethole and fenchone are major components of fennel essential oil [495,496,508], and trans-anethole is particularly noted for its significant presence and biological activities [495,508]. Trans-anethole has been shown to possess anti-inflammatory properties. It can modulate inflammatory responses by influencing cytokine production and immune cell activity [499,509,510,511]. Specifically, it has been observed to reduce IL-17 and increase IL-10 expression, which are critical in regulating inflammation [499]. Trans-anethole has been reported to inhibit the NF-κB signalling pathway, which is a key regulator of inflammation. This inhibition leads to a reduction in pro-inflammatory cytokines such as TNF-α and IL-6. This supports the claim that fennel essential oil can suppress the activation of NF-κB and reduce chronic inflammation [509,511]. This targeted approach—combining antioxidant action with the modulation of inflammatory cascades—underscores the plant’s potential as a therapeutic agent for managing inflammatory gut conditions.

In the context of a healthy gut, fennel provides a powerful, multi-faceted action: it serves as both a carminative [482,512,513] and a spasmolytic agent [496,513,514]. The trans-anethole in its essential oil is particularly effective at relaxing the smooth muscles of the gastrointestinal tract. This direct action alleviates flatulence, bloating [496,512], and abdominal pain by facilitating the expulsion of trapped gas [482,512,513]. This mechanism is especially useful for conditions like infant colic and dyspepsia, where rapid symptom relief is crucial [514].

In the in vitro study of DiNapoli et al. [489], fennel showed minimal inhibitory concentration of 250 µg·mL^−1^ against *E. coli*, *S. aureus*, *B. cereus*. Extract mixture consisted mainly of monoterpene hydrocarbons (65.64%), with αpinene (33.75%), β-pinene (5.13%), myrcene (5.25%), 3-carene (6.12%), and γ-terpinene-like (9.45%) main components. The second most abundant chemical class was that of phenylpropanoids (30.36%), with the presence of estragole (25.06%) and (E)-anethole (5.30%). Despite the weak antibacterial effect in the study, the results regarding biofilm formation inhibition are promising, as a 50% reduction in biofilm formation of *M. smegmatis* is achieved at an extract concentration of 5 µg·mL^−1^. For the other tested strains, no studies were conducted in this work. According to research Al Akeel et al. [515], as with most plant compounds, their antimicrobial effect depends on the microorganism species and the chemical composition. Extracts rich in essential oils (fenchone (24.72%), trans-anethole (22.22%), and limonene (20.48%)) studied by El Moumen et al. [516] showed the lowest MICs against *Candida albicans* (3.13 mg·mL^−1^) and *Aspergillus niger* (6.25 mg·mL^−1^), whereas higher concentrations, such as 50 mg·mL^−1^, were required to inhibit *Staphylococcus aureus* (BLACT) and *Staphylococcus epidermidis* [516]. Fennel extracts demonstrated notable antimicrobial activity, with particularly strong effectiveness against fungal pathogens, such as *Candida albicans* and *Aspergillus niger*, exhibiting MIC of 3.13 mg·mL^−1^ and 6.25 mg·mL^−1^, respectively.

Fennel has shown promising prebiotic properties, primarily due to its fibre content, particularly FOS and inulin-like compounds [516]. These non-digestible carbohydrates selectively stimulate the growth and activity of beneficial gut bacteria, such as *Lactobacillus* and *Bifidobacterium*, contributing to a healthier gut microbiota. Studies, particularly in animal models, have demonstrated the ability of fennel supplementation to positively alter faecal microbiota [517,518]. Unfortunately, there is currently a lack of direct, controlled clinical studies in humans that clearly confirm that the consumption of fennel (in the form of seeds, tea, or extracts) leads to measurable changes in the composition of the human gut microbiota typical of prebiotic activity (e.g., a significant increase in *Bifidobacterium* or *Lactobacillus*). Most available human studies focus on other properties of fennel, such as its carminative, antispasmodic effects, or its ability to alleviate dyspeptic symptoms, which may indirectly influence intestinal comfort [519].

It has also been shown to be effective on infantile colic, dysmenorrhea, polycystic ovarian syndrome and milk production [519].

A randomised, placebo-controlled trial involving 121 patients with mild-to-moderate IBS found that a 30-day treatment with a curcumin and fennel essential oil combination (CU-FEO) significantly reduced abdominal pain, improved IBS symptom scores (IBS-SSS), and enhanced quality of life [520]. These findings were consistent with a previous real-life study on 211 patients with various IBS subtypes, which also reported symptom relief and quality-of-life improvements [521]. Overall, these compounds appear to ease IBS symptoms; the fennel essential oil is thought to relax intestinal smooth muscles, reduce visceral hypersensitivity, and exert neuromodulatory effects, while curcumin contributes potent anti-inflammatory activity.

### 4.8. Garlic (Allium sativum L.)

**Figure 10:**
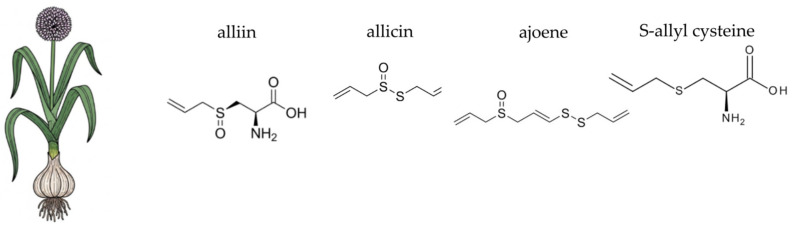
Botanical illustration of garlic and the chemical structures of selected four key bioactive compounds characteristic for this plant.

*Allium sativum* L., commonly known as garlic, is a perennial bulbous plant belonging to the *Amaryllidaceae* family (Figure 10). Native to Central Asia, it has been widely cultivated and used for millennia across many cultures for both culinary and medicinal purposes. Historical records from ancient Egyptian, Greek, Roman, Indian, and Chinese civilisations highlight its role in promoting vitality and protecting against infections [522,523]. Traditionally consumed raw or cooked as food and used in tinctures, poultices, and decoctions, garlic has more recently been formulated into standardised extracts, oils, and capsules. These preparations are often enriched in sulphur-containing compounds such as allicin, ajoene, and S-allyl cysteine, which are considered key contributors to its pharmacological effects [524,525]. Clinical and experimental studies suggest its therapeutic potential in the management of specific conditions such as hypertension, hypercholesterolemia, atherosclerosis, type 2 diabetes, and certain infectious diseases. Additionally, garlic has been investigated for its adjuvant role in the prevention of cardiovascular events and as a supportive agent in the treatment of respiratory and gastrointestinal infections [526,527,528,529]. While generally considered safe in moderate dietary amounts [523,530], concentrated garlic preparations may cause gastrointestinal discomfort [531] or allergic reactions in sensitive individuals [530], and can interact with anticoagulant medications due to its antithrombotic activity [532,533]. The following sections will detail the specific chemical components of garlic and their scientifically supported activities, with a particular focus on their contribution to gut health.

The therapeutic properties of garlic are primarily attributed to a high content of organosulphur compounds such as alliin, allicin, diallyl disulphide, and ajoene, which are responsible for its medicinal properties [534,535,536]. A key aspect of garlic’s chemistry is the release of alliin from its stable form when the plant is crushed or chopped. This process, mediated by the enzyme alliinase, produces the highly reactive and characteristic-smelling compound, allicin [537,538,539,540]. Allicin is an unstable compound that quickly breaks down into a variety of other bioactive organosulphur compounds, including diallyl trisulphide (DATS), diallyl disulphide (DADS), and ajoene, which are considered the main drivers of garlic’s pharmacological effects [536,537,539,541]. Beyond its potent sulphur compounds, garlic is a significant source of fructans, a class of non-digestible carbohydrates, including inulin and fructooligosaccharides [542,543,544]. The unique synergy between these strong antimicrobial agents and prebiotic fibres makes garlic a powerful, multifaceted agent for modulating the gut environment [51,543,545,546].

*Allium sativum* is renowned for its potent antioxidant properties, which are primarily attributed to various bioactive compounds. These compounds include organosulphur compounds, flavonoids, saponins, and polyphenols, each contributing to the antioxidant activity through different mechanisms. Allicin, an organosulphur compound, is highly reactive and exhibits significant antioxidant activity by scavenging free radicals and inhibiting lipid peroxidation. Allicin also enhances the activities of antioxidant enzymes such as superoxide dismutase and catalase, thereby reducing oxidative stress [547]. Diallyl sulphides (diallyl disulphide (DAS), DADS, DATS) inhibit oxidative damage by modulating the activity of enzymes involved in detoxification and antioxidant defence. They also exhibit pro-apoptotic and anti-inflammatory properties, contributing to their overall antioxidant effect [547,548,549]. S-allylcysteine (SAC) and S-allylmercaptocysteine (SAMC) were found in aged garlic extract (AGE); these compounds are stable and water-soluble, effectively scavenging free radicals and protecting against oxidative damage [547,549]. The polyphenolic compounds, like quercetin, gallic acid, and caffeic acid, exhibit strong antioxidant activities by directly scavenging free radicals and reducing oxidative stress. Quercetin, in particular, has been shown to significantly reduce haemolysis and oxidative damage in red blood cells [550]. Catechin, a flavonoid, is known for its role in activating peroxisome proliferator-activated receptor gamma (PPARγ), which in turn enhances the expression of antioxidant enzymes like SOD1 and CAT, thereby reducing oxidative stress [551]. Saponins in garlic contribute to its antioxidant activity by enhancing the body’s overall antioxidant capacity and protecting against lipid peroxidation [552].

*Allium sativum* exhibits significant anti-inflammatory activity due to its rich content of bioactive compounds, primarily organosulphur compounds. The key compounds responsible for this activity include allicin, ajoene, S-allylcysteine, alliin, diallyl disulphide, diallyl trisulphide, and vinyl-dithiin. These compounds exert their anti-inflammatory effects through various mechanisms. For instance, allicin inhibits pro-inflammatory cytokines such as IL1β, IL6, and IL12β, while upregulating anti-inflammatory cytokine IL10. Allicin also inhibits COX-2 activity, reducing inflammation [553]. Additionally, allicin disrupts pathogen membranes and inhibits essential enzymes through thiol group interactions [554]. Ajoene downregulates LPS-induced inflammatory cytokines and chemokines by modifying the transcription factor STAT3 through S-thiolation, which decreases its phosphorylation and nuclear translocation. Ajoene also non-competitively inhibits COX-2 activity, contributing to its anti-inflammatory properties [553]. S-allylcysteine, derived from alliin, suppresses LPS-induced inflammation by generating an anti-inflammatory gene expression profile and modifying adipocyte metabolic profiles. It decreases the phosphorylation of ERK1/2, which is involved in LPS-induced inflammation [555]. Alliin exhibits anti-inflammatory activity by binding to COX-2 enzyme with high affinity, thereby inhibiting its activity and reducing inflammation [556]. Diallyl disulphide and diallyl trisulphide contribute to the anti-inflammatory effects by modulating immune responses and reducing cytokine levels [557,558]. Vinyl-dithiin has neuroprotective and anti-inflammatory properties, although the specific mechanisms are less well-documented compared to other sulphur compounds [557]. This comprehensive approach—combining direct antioxidant action with the modulation of inflammatory cascades—underscores the plant’s potential as a therapeutic agent for managing inflammatory gut conditions.

Allicin, though highly reactive and chemically unstable, is regarded as the primary antibacterial constituent of garlic. Other sulphur-rich compounds—such as diallyl sulphide, diallyl disulphide, diallyl trisulphide, and various polysulphides—also contribute significantly to garlic’s antimicrobial properties. Their antibacterial efficacy generally increases with the number of sulphur atoms (DAS_4_ > DAS_3_ > DAS_2_ > DAS_1_) [559]. These compounds exert their effects by covalently binding to free sulphydryl (-SH) groups in bacterial proteins and essential metabolic enzymes, resulting in irreversible inhibition of targets such as urease, alcohol dehydrogenase, and proteases [536]. In bacterial pathogens, plant-derived essential oils compromise the integrity of the cell membrane, leading to the leakage of intracellular components and the loss of vital ions. This disruption interferes with essential cellular processes such as energy production and membrane transport, ultimately resulting in bacterial cell death [536,560,561].

Diallyl disulphide, a major constituent of garlic essential oil (comprising 20–30% of its content), has been shown to affect *Bacillus cereus* by extending the lag phase, lowering the maximum bacterial population, and suppressing key virulence determinants. Importantly, it also reduces toxin production and biofilm formation even at sub-inhibitory concentrations (≤60 μg·mL^−1^) [562]. In vitro study suggests that such garlic-derived formulations may hold therapeutic potential as adjunct or alternative treatments for *H. pylori* infections, potentially reducing or eliminating the need for proton pump inhibitors during clinical management. Solvent-free garlic extracts prepared with ethanol and acetone effectively inhibited *H. pylori* growth in vitro under simulated gastric pH and body temperature conditions. The antimicrobial activity was pH-dependent, with maximal inhibition observed at pH 2.5 and a notable decline below pH 2.0, likely due to protonation of the sulfinyl-sulphur oxygen, which reduces thiosulphinate (allicin) levels [563].

Garlic compounds also show efficacy in reducing biofilm formation, inhibiting toxin secretion, and enhancing antibiotic efficacy in synergy studies [564]. Additionally, studies have shown that allyl sulphides present in garlic essential oil exert antibacterial effects by modulating the expression of genes involved in bacterial metabolism, membrane transport, and secretion systems. For instance, in *Campylobacter jejuni*, allyl sulphide downregulates 14 genes associated with cellular homeostasis and oxidative stress response, thereby impairing the bacterium’s ability to adapt to hostile environmental conditions [565]. Study on *Pseudomonas aeruginosa* in vivo and in vitro showed suppression of the expression of *las*, *rhl*, and *pqs* by addition of the allyl sulphide in garlic essential oil [566].

In the context of a healthy gut microbiome, garlic provides a powerful dual action: it functions as both a prebiotic and an antimicrobial agent. The fructans in garlic, including inulin and FOS, serve as a non-digestible dietary fibre that reaches the colon intact. Here, they are selectively fermented by beneficial gut bacteria, primarily species of *Bifidobacteria* and *Lactobacillus*. This fermentation process yields short-chain fatty acids, particularly butyrate, which is a vital energy source for colonocytes and plays a critical role in maintaining the integrity of the intestinal barrier [567]. Furthermore, the organosulphur compounds, especially allicin, possess broad-spectrum antimicrobial properties. They can inhibit the growth of pathogenic bacteria, fungi, and yeasts, including *Helicobacter pylori* and *Candida albicans*, without a substantial negative impact on the beneficial commensal flora [536,559]. This unique combination of supporting beneficial bacteria through prebiotic fibre while simultaneously inhibiting pathogens makes garlic a potent and well-rounded agent for promoting overall gut homeostasis.

A particularly interesting study was conducted in China—the Shandong Intervention Trial, a long-term, randomised intervention trial that examined the effects of vitamin and garlic supplementation, as well as *H. pylori* treatment, on the risk of gastric cancer in a high-risk population. The study showed that garlic supplementation resulted in a statistically significant reduction in gastric cancer mortality and a promising, though not statistically significant, reduction in gastric cancer incidence [568].

The study of Rahmatinia et al. [569] showed that garlic supplementation was effective in lowering blood pressure, improving lipid profile, and increasing nitric oxide levels in prehypertensive participants [569]. According to the results of a meta-analysis by Fu et al. [570], garlic appears to have a modulatory effect on certain components of metabolic syndrome [570]. After 6 weeks, consumption of 250 mg of aged black garlic (ABG) extract containing 1.25 mg of SAC significantly reduced diastolic blood pressure in subjects with moderate hypercholesterolemia [571].

### 4.9. Ginger (Zingiber officinale Roscoe)

**Figure 11:**
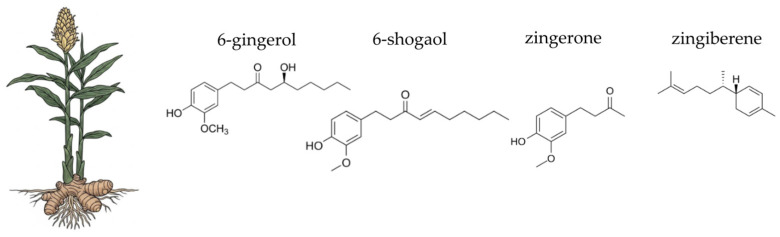
Botanical illustration of ginger and the chemical structures of selected four key bioactive compounds characteristic for this plant.

Ginger (*Zingiber officinale* Roscoe), a flowering perennial plant of the *Zingiberaceae* family, is widely cultivated for its rhizome, which is used both as a spice and in traditional medicine (Figure 11). Indigenous to Southeast Asia, ginger has been domesticated and utilised for thousands of years across various cultures, including those of India, China, and the Middle East, where it was esteemed for its culinary versatility and its reputed role in alleviating digestive discomfort [572,573]. Traditionally consumed as a fresh or dried rhizome, it is also prepared as powders, teas, tinctures, or incorporated into food and beverages [574,575]. In modern applications, ginger is increasingly available in standardised extract forms, although whole root preparations remain common [576]. Ginger is used to treat various ailments including nausea, vomiting, gastrointestinal disorders, and respiratory conditions [577,578,579]. Ginger exhibits anticancer properties, with compounds like [6]-gingerol and [6]-shogaol playing a crucial role in inhibiting cancer cell growth and inducing apoptosis [580,581]. Ginger has also potential neuroprotective effects, which may be beneficial in treating neurodegenerative diseases such as Alzheimer’s and Parkinson’s disease due to its ability to modulate oxidative stress and neuroinflammation [582,583]. The plant is recognised for its pungent aroma and warming taste, which have contributed to its widespread culinary adoption globally [584]. Ginger is generally regarded as safe for most individuals when consumed in moderate dietary amounts [585]. However, given the potential for ginger to affect platelet function, individuals with bleeding disorders or those on anticoagulant therapy should be cautious with ginger consumption. The antiplatelet effects of ginger could exacerbate bleeding tendencies or interact with anticoagulant medications, increasing the risk of bleeding complications [586]. The following sections will detail the specific chemical components of ginger and their scientifically supported activities, with a particular focus on their contribution to gut health.

The pharmacological properties of ginger are primarily attributed to a complex mixture of non-volatile and volatile compounds. Ginger contains several phenolic compounds such as gingerols and shogaols. [6]-gingerol is the primary active compound responsible for the characteristic pungency of fresh ginger [580]. When ginger is dried or heated, gingerols are converted into shogaols, with [6]-shogaol being more pungent and pharmacologically active [577,582]. The aroma of ginger is primarily due to essential oils, which are rich in sesquiterpenes like zingiberene [422,587]. The conversion of gingerols to shogaols upon drying or heating enhances the pungency and bioactivity of ginger. This transformation is significant as shogaols exhibit stronger pharmacological effects compared to gingerols [577,582]. The combination of gingerols/shogaols and terpenes creates a synergistic effect that underpins ginger’s therapeutic properties. This synergy contributes to ginger’s wide range of pharmacological activities, including anti-inflammatory, antioxidant, antimicrobial, anticancer, and neuroprotective effects [422,583,588,589].

The antioxidant activity of *Zingiber officinale* is attributed to several bioactive compounds, each with distinct mechanisms of action, for example, compounds such as [6]-gingerol, [8]-gingerol, and [10]-gingerol exhibit significant antioxidant activity by scavenging free radicals like DPPH, superoxide, and hydroxyl radicals. They also inhibit reactive oxygen species production in human polymorphonuclear neutrophils and reduce the production of inflammatory mediators such as nitric oxide and prostaglandin E2 [590,591,592,593]. The carbon chain length influences their potency, with [10]-gingerol [592] being the most effective among the gingerols [593]. [6]-shogaol, another potent antioxidant, demonstrates strong free radical scavenging activity and inhibits ROS production. Its effectiveness is attributed to the presence of an α,β-unsaturated ketone moiety [590,593]. Other compounds, like zingerone and dehydrozingerone, derived from ginger rhizomes, show potent antioxidant activities. Dehydrozingerone and its synthetic analogues inhibit Fe^2+^-induced lipid peroxidation and exhibit radical scavenging activity comparable to standard antioxidants like α-tocopherol and ascorbic acid [594]. The butanol fraction of ginger, rich in phenolic compounds, shows strong antioxidant potential by reducing oxidative stress markers such as TNF-α and MDA, and by maintaining CAT activity and glutathione (GSH) levels [595]. Flavonoids contribute to ginger’s antioxidant activity by scavenging free radicals and reducing blood glucose levels. They are particularly effective when extracted using natural deep eutectic solvents (NADES) [596]. The essential oil of ginger, containing compounds like benzene, camphene, and 1R-alpha pinene, exhibits concentration-dependent antioxidant activity [597,598]. Polyphenols, including gingerols and shogaols, are primarily responsible for ginger’s antioxidant properties. They act by scavenging free radicals, inhibiting lipid peroxidation, and modulating antioxidant enzyme activities [598].

*Zingiber officinale*, commonly known as ginger, exhibits significant anti-inflammatory activity due to several bioactive compounds, primarily gingerols and shogaols. These compounds act through various mechanisms to exert their anti-inflammatory effects. [6]-shogaol, [6]-gingerol, [8]-gingerol, [10]-gingerol demonstrate substantial free radical scavenging activities, inhibiting the production of reactive oxygen species and inflammatory mediators such as NO and PGE2 in human polymorphonuclear neutrophils and RAW 264.7 cells [593]. The presence of α,β-unsaturated ketone moiety in [6]-shogaol contributes to its potent antioxidant and anti-inflammatory properties [593,598,599]. [6]-shogaol, [6]-gingerol, [8]-gingerol, and [10]-gingerol are compounds that inhibit protein denaturation, membrane stabilisation, protease activity, and lipoxygenase activity, which are crucial in the inflammatory process. In vivo studies show that aqueous extracts containing these compounds reduce pro-inflammatory cytokines such as TNF-α, IL-6, and monocyte chemoattractant protein-1 (MCP-1), and increase total antioxidant capacity [599]. Novel compounds such as diphenylheptane dimers found in ginger peel exhibit effective anti-inflammatory activity by reducing the production of iNOS and other inflammatory markers in LPS-induced RAW 264.7 cells [600]. Ginger’s phenolic compounds, including gingerols and shogaols, influence the function of immune cells such as macrophages, neutrophils, dendritic cells, and T cells. These compounds target multiple signalling pathways, including the inhibition of pro-inflammatory cytokines and enhancement of antioxidant performance [583].

In the context of gut health, one of ginger’s most notable actions is its direct influence on gastrointestinal motility via inhibition of serotonin 5-HT_3_ receptors [601]. [6]-gingerol and [6]-shogaol have been shown to stimulate the smooth muscles of the gastrointestinal tract. This effect is observed both in vitro and in vivo, where ginger attenuates acetylcholine-induced contractions in the small intestine and colon [602,603,604]. This prokinetic effect promotes gastric emptying and accelerates the movement of food through the intestines, which is particularly beneficial for alleviating dyspepsia, bloating [605,606], and nausea. This mechanism, along with its anti-inflammatory properties, makes ginger a primary therapeutic choice for symptoms like morning sickness [607].

Numerous in vitro studies have confirmed the antimicrobial activity of ginger essential oil, extracts, and oleoresins against various bacteria and fungi. This activity is largely attributed to phenolic compounds such as gingerols, shogaols, and eugenol, as well as their synergistic effects with terpenes like β-sesquiphellandrene and α-farnesene. The efficacy depends on factors such as chemical composition, extraction method, and solvent used, with organic extracts showing greater activity than aqueous ones due to better solubility of active compounds [608]. The study [609] highlights that [6]-gingerol, a prominent compound in ginger, disrupts bacterial cell membranes and efflux pumps, leading to reduced virulence and biofilm formation in both *Staphylococcus aureus* and *Pseudomonas aeruginosa*. It also notes [6]-gingerol’s ability to inhibit quorum sensing. The research screened various ginger extracts against *Streptococcus mutans* and *Streptococcus sobrinus*, identifying polyphenols, alkaloids, flavonoids, and terpenoids as active constituents. Methanol and ethyl acetate extracts showed the highest antibacterial activity, with compounds demonstrating good binding affinity to target enzymes in *S. mutans* [610].

Ginger extract (GIN) exhibited a stronger stimulatory effect than cinnamon, especially on *Lactobacillus* spp. At 1.13 mg·mL^−1^, it enhanced the growth of 7 strains, and at 2.25 mg·mL^−1^, 4 strains responded positively [443]. GIN also promoted the growth of *L. reuteri* and *L. rhamnosus*, aligning with previous findings on its ability to support microbial balance in the gastrointestinal tract [443,444]. While its effect on *Bifidobacterium* spp. was moderate and comparable to that of cinnamon and black pepper, the overall response suggests that ginger may act as a prebiotic-like compound by stimulating the growth of beneficial gut bacteria.

Supplementation with 3 g per day of ginger powder in patients with *H. pylori*-positive functional dyspepsia resulted in significant eradication of *H. pylori* and improvement in dyspeptic symptoms. However, this study involved only 15 patients [611].

A clinical study in 56 patients with clinically diagnosed IBD demonstrated that gingerol from *Zingiber officinale* (730 mg daily for 30 days as a herbal supplement) enhanced the efficacy of spasmolytics, laxatives, and/or proton pump inhibitors [612].

Ginger exhibits both spasmolytic and spasmogenic properties through inhibition of cholinergic responses and K^+^-induced contractions, as well as reduction of colonic oedema and inflammation via NF-κB modulation [613]. Treatment with an herbal mixture containing ginger has been associated with decreased severity and frequency of abdominal pain, reduced bloating, and improvements in depression and anxiety scores in affected patients [614].

### 4.10. Green Tea (Camellia sinensis (L.) Kuntze)

**Figure 12:**
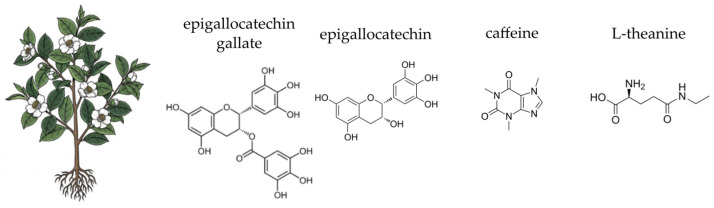
Botanical illustration of green tea and the chemical structures of selected four key bioactive compounds characteristic for this plant.

Green tea (*Camellia sinensis* (L.) Kuntze) is an evergreen shrub of the *Theaceae* family, it is native to East Asia and has been cultivated and consumed for millennia (Figure 12). It is deeply rooted in traditional Chinese, Japanese, and other Asian cultures for both culinary and medicinal use [615,616,617,618]. Traditionally prepared as an infusion from minimally oxidised leaves, green tea is renowned for its health-promoting properties [619]. It is widely consumed worldwide today, available as dried leaves, tea bags, and concentrated extracts in dietary supplements [620]. Green tea has been extensively studied for its beneficial effects in the prevention and management of various conditions, including cardiovascular diseases [621], metabolic disorders such as type 2 diabetes [622], neurodegenerative diseases [623], and certain types of cancer [624]. It also shows potential in supporting weight management [625] and improving digestive health [626]. Generally regarded as safe for human consumption, caution is advised when consuming high doses of concentrated green tea extracts, due to rare but reported cases of liver toxicity [627]. The following sections will detail the specific chemical components of green tea and their scientifically supported activities, with a particular focus on their contribution to gut health.

The therapeutic properties of green tea are primarily attributed to a class of polyphenolic compounds known as catechins. Among these, epigallocatechin gallate (EGCG) is the most abundant and pharmacologically significant, constituting approximately 50–60% of the flavonoids in fresh green tea leaves [628,629]. EGCG is celebrated for its potent antioxidant, anti-inflammatory, cardioprotective, and antitumor properties, making it a key driver of green tea’s health benefits [628,630,631,632,633]. Other important catechins include epicatechin (EC), epigallocatechin (EGC), and epicatechin gallate (ECG). Unlike black tea, green tea undergoes minimal oxidation, which preserves its high catechin content. This minimal processing is crucial for maintaining the therapeutic properties of green tea catechins [629]. Despite the promising health benefits, the clinical use of green tea catechins, particularly EGCG, is constrained by issues such as poor bioavailability and stability. Various encapsulation techniques, including nanoparticles and lipid nanocapsules, have been explored to enhance the bioavailability and efficacy of these compounds [632,634,635]. In addition to catechins, green tea contains other important bioactive compounds, including flavonoids like quercetin and kaempferol [636,637], phenolic acids like gallic acid [638], and a small amount of caffeine [639]. The synergistic action of these various components, with EGCG at the forefront, underpins green tea’s potent antioxidant and anti-inflammatory effects. The unique chemical profile of green tea, particularly its high EGCG content, makes it a powerful agent for modulating the gut environment and supporting overall digestive health [629,633,637,640,641].

*Camellia sinensis* contains several compounds responsible for its antioxidant activity, each with distinct mechanisms of action. The primary bioactive compounds include catechins, flavonoids, phenolic acids, and polysaccharides. EGCG is a potent antioxidant that inhibits enzymes involved in cholesterol and uric acid metabolism, such as hydroxy-3-methyl-glutaryl-CoA reductase and xanthine oxidase. It also affects glucose transporters, contributing to its pharmacological effects [640,642]. EGCG exhibits strong radical scavenging activity, reducing oxidative stress and protecting cells from damage [642,643,644]. EC, EGC, GCG, and GC contribute to the antioxidant activity by neutralising free radicals and inhibiting lipid peroxidation [645]. Their structural features, such as the presence of hydroxyl groups, enhance their redox properties [640]. Quercetin is another significant antioxidant found in *Camellia sinensis*. It exhibits high chemical stability and reactivity, contributing to its ability to scavenge free radicals and inhibit oxidative stress. Quercetin also shows antibacterial activity, which may be linked to its antioxidant properties [642]. Caffeic acid is known for its antioxidant properties, including the ability to scavenge free radicals and inhibit lipid peroxidation. Caffeic acid contributes to the overall antioxidant capacity of *Camellia sinensis* extracts [642]. Tea polysaccharides (TPS) are bioactive constituents that exhibit antioxidant and anti-inflammatory effects. They inhibit digestive enzymes, prevent macronutrient absorption, and modulate gene and protein expression related to oxidative stress. TPSs also play a role in gut health, which can indirectly affect antioxidant activity by maintaining a healthy microbiome [646].

*Camellia sinensis* contains several bioactive compounds responsible for its anti-inflammatory activity. The primary compounds include catechins, flavonoids, phenolic acids, and saponins, each contributing through various mechanisms. The most potent catechin is epigallocatechin gallate, which exhibits significant anti-inflammatory effects by neutralising reactive oxygen species and inhibiting pro-inflammatory cytokines such as TNF-α and IL-6 [642,647,648]. EGCG also suppresses the nuclear NF-κB pathway, reducing the expression of inflammatory mediators like iNOS [649]. Other catechins, such as EC, EGC, and ECG, also contribute to anti-inflammatory activity by similar mechanisms [647,648]. Quercetin, a flavonoid found in *Camellia sinensis*, has been shown to inhibit the production of inflammatory cytokines and reduce oxidative stress by stabilising the NF-κB pathway. This compound also exhibits high chemical stability, enhancing its therapeutic potential [642,650,651]. Caffeic acid and syringic acid are phenolic acids that contribute to its anti-inflammatory properties. These compounds inhibit the production of ROS and reduce lipid peroxidation, thereby mitigating inflammation [642,652]. Theaflavin, a saponin found in *Camellia sinensis*, has demonstrated high affinity in molecular docking studies for anti-apoptotic and anti-pyroptotic activities, which are crucial in reducing inflammation [653]. Another saponin, 21-O-angeloyltheasapogenol E3 (ATS-E3), inhibits the AKT/IKK/NF-κB signalling pathway, further contributing to its anti-inflammatory effects [649]. This comprehensive approach—combining direct antioxidant action with the modulation of inflammatory cascades—underscores the plant’s potential as a therapeutic agent for managing inflammatory gut conditions.

Furthermore, green tea catechins play a critical role in strengthening the intestinal barrier. EGCG has been shown to enhance the expression of genes responsible for maintaining tight junctions between intestinal epithelial cells. Tight junction proteins such as claudin-1, claudin-4, and occludin are upregulated by EGCG, which helps in stabilising the intestinal barrier and preventing barrier dysfunction [654,655]. EGCG has been shown to protect against barrier defects induced by pro-inflammatory cytokines like TNFα and IFNγ. This protection is evidenced by increased transepithelial resistance (TER) and reduced macromolecular permeability, indicating a stronger barrier function [654]. A strong intestinal barrier is vital for preventing the translocation of toxins, undigested food particles, and pathogens into the bloodstream, which can trigger systemic inflammation [347,656]. This combination of supporting a healthy microbial balance and reinforcing the gut barrier makes green tea a potent and well-rounded agent for promoting overall gut homeostasis and resilience [657,658,659].

The main antimicrobial compounds in green tea are catechins [660]. Epigallocatechin gallate is the most abundant and biologically potent catechin in green tea, followed by epicatechin gallate and epigallocatechin. Catechins, especially EGCG, can directly bind to and damage bacterial cell membranes, leading to increased permeability, leakage of cytoplasmic contents, and ultimately cell death. This is particularly effective against Gram-positive bacteria [661]. Catechins can inhibit various bacterial enzymes essential for growth, metabolism, and virulence. For instance, EGCG has been shown to inhibit dihydrofolate reductase in some bacteria green tea also contains other flavonoids (e.g., quercetin, kaempferol, myricetin) and phenolic acids (e.g., gallic acid, caffeic acid, chlorogenic acid) that may contribute to its overall antimicrobial spectrum [662].

Green tea has been increasingly recognised for its prebiotic potential, characterised by its ability to selectively enhance the growth and metabolic activity of beneficial gut microbiota. Although it does not contain traditional prebiotic fibres, its polyphenolic constituents—primarily catechins such as epigallocatechin gallate—undergo biotransformation by intestinal microbes, serving as substrates that support microbial proliferation. This interaction contributes to favourable modulation of the gut microbiome, influencing both microbial composition and host metabolic functions [663]. A large portion of green tea catechins are not absorbed in the small intestine due to their complex structure and low bioavailability. Instead, they reach the colon where they are fermented by gut bacteria. This fermentation process selectively promotes the growth of beneficial bacteria (e.g., *Bifidobacterium*, *Lactobacillus*, *Akkermansia muciniphila*) [475,664], while often inhibiting the growth of less desirable or pathogenic bacteria (e.g., *Clostridium species*, certain *Enterobacteriaceae*). Gut bacteria metabolise green tea polyphenols into various smaller, more bioavailable phenolic acids and other metabolites (e.g., short-chain fatty acids like butyrate) [665]. These metabolites contribute to gut health, influence host metabolism, and can have systemic anti-inflammatory and antioxidant effects [657,666,667].

Green tea is associated with improved cardiovascular outcomes. Its catechins can help reduce LDL cholesterol and total cholesterol levels, improve endothelial function, and contribute to modest reductions in blood pressure. These effects are attributed to its antioxidant, anti-inflammatory, and anti-thrombogenic properties. Regular green tea consumption has been linked to a reduced risk of cardiovascular disease mortality.

A meta-analysis of 59 studies involving 3802 participants showed that green tea extract (GTE) supplementation significantly reduced body mass, body fat percentage, BMI, and MDA, while increasing adiponectin and TAC, with no effects on fat mass, leptin, or ghrelin. The certainty of evidence ranged from low to high. Overall, GTE appears to reduce oxidative stress and improve body composition markers, while enhancing antioxidant status and adiponectin levels [668].

Separately, Colonetti et al. [669] conducted a double-blind RCT in 169 women with PCOS, with 85 in the green tea group and 84 in the placebo group. The green tea group showed significantly lower body weight (mean difference: −2.80 kg). The authors concluded that green tea may help reduce weight, but further studies are needed to confirm its effect and support its use as an adjuvant treatment in PCOS management [669].

### 4.11. Summary

The table below (Table 2) synthesises key information regarding ten medicinal plants with a documented impact on the gut microbiome and overall gastrointestinal health. The assessment of activity strength (weak, moderate, strong, very strong) is a relative evaluation based on available scientific evidence, including in vitro, in vivo, and clinical studies.

## 5. Limitations and Considerations

While medicinal plants offer a promising approach to supporting gut health, their application is subject to several key limitations and requires careful consideration. The efficacy of herbal treatments can be influenced by a range of factors that are often overlooked in both traditional use and modern research.

A primary challenge lies in the bioavailability of phytochemicals. Many of the bioactive compounds discussed in this article, such as polyphenols and triterpenoids, have poor aqueous solubility and are susceptible to degradation by stomach acid and digestive enzymes [694,695]. Their absorption and subsequent metabolic transformation by the liver can be highly inefficient. This transformation often results in the formation of metabolites that may have reduced or altered bioactivity [696].

A further critical aspect is the pronounced inter-individual variability in host pharmacology, driven by the gut microbiome. The human gut microbiota is highly unique, shaping the pharmacokinetics of ingested phytochemicals. As a result, the same herbal compound can be metabolised differently, leading to variable systemic exposure and divergent therapeutic outcomes [697,698,699]. This presents a significant practical challenge for the herbs discussed. For instance, the clinical efficacy of green tea catechins (e.g., epigallocatechin gallate) is highly dependent on an individual’s “metabotype” [628,630]. The biotransformation of EGCG into more bioavailable secondary metabolites, such as valerolactones, is conducted by specific microbial pathways (e.g., involving *Eggerthella lenta*), which are absent in a significant portion of the population [700,701,702,703]. This creates a ‘non-responder’ phenotype, where individuals may not experience the anticipated metabolic benefits. A similar challenge exists for garlic; its primary bioactive compound, allicin, is unstable and its metabolism into various organosulphur compounds is heavily influenced by the gut microbiota, thereby altering clinical efficacy in lipid modulation [704,705]. Furthermore, the prebiotic potential of plants like dandelion and globe artichoke, rich in inulin-type fructans, is entirely contingent on the host possessing a robust population of saccharolytic bacteria (e.g., *Bifidobacterium* and *Faecalibacterium*) capable of fermenting them into beneficial short-chain fatty acids [706,707,708]. In individuals lacking these specific functional guilds, supplementation may fail to yield therapeutic benefits and could instead induce adverse gastrointestinal symptoms, such as bloating and flatulence. This biological variability can make it challenging to predict the efficacy of a particular herbal treatment and complicates the standardisation of care. It also highlights the need for a more personalised approach to herbal medicine, where the composition of an individual’s gut microbiota is considered in treatment selection [564,709].

Another critical consideration is the complex pharmacodynamics related to dosage and preparation. The concentration of active compounds can differ dramatically depending on the plant part used (e.g., root vs. leaf) [710], cultivation conditions [711,712], harvesting time [711,713], and the processing methods (e.g., drying, extraction) [712,714]. Standardised extracts are designed to mitigate this issue by guaranteeing a specific concentration of a key compound (e.g., silymarin from milk thistle), but they may lack the synergistic effects of the whole plant matrix [715]. Furthermore, clinical application is complicated by the distinct, dose-dependent and biphasic effects observed for several of these plants. A salient example is aloe vera; at low doses, the inner leaf gel is widely used for its soothing, mucilaginous (polysaccharide-driven) properties for the gastric mucosa, whereas at higher doses, the anthraquinones (e.g., aloin) present in the outer leaf latex exert a potent stimulant laxative effect [231,278,716,717,718]. Similarly, the therapeutic actions of ginger appear to be dose-specific: low-to-moderate doses (e.g., 1–2 g per day of powder) are clinically validated for managing nausea, while the higher doses (e.g., >3 g per day) required for systemic anti-inflammatory effects (driven by gingerols and shogaols) carry a different benefit-risk profile [583,719,720,721,722]. This concept extends to Ceylon cinnamon. While moderate doses are studied for glycaemic control, higher doses (or the use of *C. cassia* species) can introduce clinically significant levels of coumarin, a potential hepatotoxin, highlighting a narrow therapeutic window [393,395,404,723,724]. This highlights that ‘efficacy’ is not a single measure but is tied directly to a specific dose for a specific indication, complicating the use of whole-plant forms where ensuring consistent dosing remains challenging [669,725,726].

Moreover, the risk of contaminants is a significant concern. Medicinal plants can contain pesticide residues and absorb heavy metals such as lead, cadmium, chromium, and mercury from contaminated soil, water, and air [727,728,729,730,731]. They are also susceptible to microbial contamination, particularly with mould, fungi, and bacteria, which can be introduced during harvesting, drying, or storage [730,732]. The market for medicinal plants is often unregulated, leading to inconsistent quality and safety standards. Regular monitoring and inclusion of medicinal plants in appropriate regulatory frameworks are necessary to ensure their safety and purity [730,733].

Finally, the lack of robust clinical data for many traditional herbal applications poses a limitation. While in vitro and animal studies provide valuable insights into the mechanisms of action, well-designed, large-scale human clinical trials are often sparse [734,735,736]. Research on herbal applications for complex conditions, such as inflammatory bowel disease, often faces challenges in standardising dosage, ensuring patient compliance, and measuring long-term outcomes [737,738]. This knowledge gap makes it difficult to establish clear clinical guidelines and may limit the integration of herbal therapies into mainstream medical practice. Ensuring patient compliance is another challenge. Observational studies and non-interventional studies can help gather data, but they may not always meet regulatory requirements or provide high-quality documentation [735,739].

## 6. Materials and Methods

The ten medicinal plants forming the basis of this review (globe artichoke, aloe vera, German chamomile, pot marigold, Ceylon cinnamon, dandelion, fennel, garlic, ginger, and green tea) were purposefully selected prior to the literature search. The selection was based on a preliminary scoping of the field and required each plant to meet three primary criteria: (i) a well-documented history of traditional use in managing digestive ailments; (ii) global relevance and widespread availability as either a food ingredient or herbal medicine; and (iii) the existence of a substantial body of modern scientific research indicating potential bioactivity within the gastrointestinal tract. This approach ensures that the review focuses on plants whose historical applications are now being investigated through contemporary scientific methods.

This review was conducted following a systematic approach to ensure a comprehensive and unbiased selection of literature. A systematic search was performed across multiple electronic databases, including PubMed, Scopus, and Web of Science, to identify relevant articles published between January 1992 and September 2025. This broad temporal scope was intentionally chosen to ensure a comprehensive overview of the topic, encompassing both foundational studies and the most recent scientific advancements in the field.

The search strings were developed using a structured combination of keywords to ensure reproducibility. The strategy involved combining terms related to each specific medicinal plant (Concept A) with a broad set of terms related to the gut microbiome and digestive health (Concept B), linked by the Boolean operator “AND”. Within each concept, synonyms and related terms were combined using the “OR” operator.

The general structure of the search query was as follows: (Concept A: plant name OR latin name OR key bioactive compound) AND (Concept B: “gut microbiome” OR “gut microbiota” OR “intestinal flora” OR “dysbiosis” OR “prebiotic” OR “digestive health”).

This search template was adapted and executed for each of the ten plants covered in this review. To further enhance the comprehensiveness of the search, a manual search of the reference lists of all included articles was also conducted to identify additional relevant publications.

The identified articles were then screened in a two-stage process. First, two independent reviewers screened all articles based on their titles and abstracts to remove irrelevant studies. Any disagreements at this stage were resolved through discussion. Second, the full texts of the remaining articles were retrieved and assessed against the following pre-defined eligibility criteria: (1) inclusion criteria: (i) peer-reviewed articles published in the English language; (ii) original research (including in vitro, in vivo, and clinical studies) or meta-analyses; (iii) studies focusing on one of the ten pre-specified medicinal plants and their effect on the gut microbiome or relevant digestive health markers; (2) exclusion criteria: (i) narrative reviews, editorials, conference abstracts, and book chapters; (ii) studies on multi-herb formulations where the effect of a single plant could not be isolated; (iii) research where the primary focus was unrelated to gastrointestinal or microbiome-related outcomes.

## 7. Conclusions and Future Perspectives

This review has highlighted the profound and multifaceted role of selected medicinal plants in promoting a healthy gut microbiome. As demonstrated, medicinal plants do not exert their effects through a single mechanism. Instead, they act synergistically via a diverse range of bioactive compounds—including polyphenols, flavonoids, polysaccharides, and essential oils—to modulate the gut environment. Their beneficial actions can be broadly categorised into three key areas: (1) Antioxidant and anti-inflammatory effects: by neutralising reactive oxygen species and inhibiting pro-inflammatory cytokines like TNF-α and IL-6, these plants help to protect the intestinal barrier from damage and mitigate chronic, low-grade inflammation. (2) Prebiotic and antimicrobial properties: many of these plants, particularly dandelion and globe artichoke (rich in inulin) and garlic (rich in fructans), provide fermentable carbohydrates that selectively nourish beneficial gut bacteria. Simultaneously, compounds like cinnamaldehyde and allicin exhibit antimicrobial activity, helping to inhibit pathogens and restore microbial balance. (3) Direct influence on gut function: plants such as fennel and ginger directly impact gut motility and alleviate digestive discomfort through their spasmolytic and carminative actions, while pot marigold and aloe vera promote mucosal healing and barrier integrity.

Given their complex mechanisms of action, medicinal plants represent a valuable, yet often underutilised, tool in modern gastroenterology. Their ability to address multiple pathways simultaneously—from reducing inflammation and oxidative stress to supporting microbial balance—makes them particularly well-suited for the management of multifactorial gut disorders. The future of phytotherapy lies in its integration with personalised medicine. Rather than a one-size-fits-all approach, a tailored strategy that considers an individual’s unique gut microbiome composition, genetic predispositions, and dietary habits could significantly enhance therapeutic outcomes. By utilising microbiota biomarkers and advanced diagnostic tools, clinicians could select specific plant-based treatments that are most likely to be effective for a given patient’s gut profile, thereby optimising their health and wellbeing.

Despite the wealth of traditional knowledge and promising preclinical (in vitro and animal) data, translating this evidence into routine clinical practice presents several critical challenges. The gap between experimental findings and clinical application must be bridged before these plants can be responsibly integrated into patient care. Future efforts must focus on: (1) Standardisation and quality control: A significant barrier to clinical adoption is the profound lack of standardisation. As highlighted in the limitations, the phytochemical profile of a plant (e.g., allicin content in garlic or cinnamaldehyde in cinnamon) varies dramatically based on cultivation, processing, and extraction methods. Without robust standardisation and good manufacturing practices (GMP), it is impossible to ensure a consistent, reproducible dose, rendering clinical trial comparisons and reliable patient outcomes unattainable. (2) Pharmacokinetics and drug-plant interactions (DPIs): This is perhaps the most critical clinical challenge. Patients with gut disorders are often taking multiple conventional medications (e.g., proton pump inhibitors, immunosuppressants, or biologics). Phytochemicals from plants like green tea (EGCG) or ginger are known to modulate crucial metabolic pathways, particularly the cytochrome P450 enzyme system in the liver. This creates a high risk of DPIs, potentially altering the efficacy or increasing the toxicity of co-administered drugs. Rigorous pharmacokinetic studies are urgently needed to establish safety profiles and clear clinical guidelines. (3) Designing robust clinical trials: Moving beyond preclinical models requires large-scale, placebo-controlled human trials. However, these face unique methodological hurdles, such as difficulties in effectively blinding interventions (due to the distinct taste and smell of herbs like fennel or garlic) and ensuring patient compliance with complex, long-term herbal regimens.

In conclusion, medicinal plants offer a compelling and sustainable avenue for supporting the gut microbiome. By addressing the current limitations through rigorous scientific inquiry, we can unlock their full therapeutic potential and pave the way for a new era of personalised phytotherapy.

## Figures and Tables

**Table 1:** Occurrence of different types of probiotics in selected medicinal plants.

Types of Prebiotics	Occurrence in Selected Medicinal Plants	Potential Benefits	Reference
Fructooligosaccharides (FOS)	artichoke, garlic—75% of its dry weight, dandelion, artichoke, chamomile, aloe vera	Improve calcium absorption, decrease triglycerides, improve immunity, inhibit pathogenic microorganisms, prevent cancer, and control diabetes	[52,53,54]
Galactooligosaccharides (GOS)	lack of this compound in plants	Increase bifidogenic activity	[55,56]
Xylooligosaccharides (XOS)	no	Non-carcinogenic nature, exhibit a positive effect on the intestinal flora, non-digestibility	[57]
Soybean oligosaccharides (SOS)	no	Increase the level of IgG, modulate body weight and the immune system	[58]
Isomaltooligosaccharides (IMO)	no	Improve gastrointestinal flora	[59]
Fructans	dandelion—40% of dry matter, artichoke, aloe vera, chamomile, garlic	Modulate gut physiology to provide protection from pathogens, improve the level of glucose,	[60,61,62]
Guar gum	no	Improve cholesterol, glycemia	[57,63]
Pectinoligosaccharides (POS)	pot marigold	Anti-inflammatory effect	[64]

**Table 2:** A comparative assessment of the bioactivity of selected medicinal plants.

Plant Name (Latin Name)	Key Active Compounds	Plant Part Used	Examples of Dosages	Antioxidant Activity	Anti- Inflammatory Activity	Antimicrobial Activity	Prebiotic Effect	References
Globe artichoke (*Cynara scolymus*)	cynarin, chlorogenic acid, luteolin, apigenin, fructooligosaccharides, inulin-type fructans	leaves, flower heads	- infusion: 2–4 g of dried leaves per 250 mL of water, 2–3 times daily - extract: 300–600 mg of a standardised extract, 2–3 times daily	strong	moderate	weak	very strong	[61,184,670,671,672]
Aloe vera (*Aloe vera*)	acemannan, aloin, aloesin, fructooligosaccharides, fructans	leaf pulp (gel)	- juice/gel: 30–50 mL, 2–3 times daily - extract: 100–200 mg of dry extract daily	moderate	strong	moderate	strong	[673,674,675,676]
German chamomile (*Matricaria chamomilla*)	α-bisabolol, luteolin, apigenin, quercetin, patuletin, fructooligosaccharides	flower heads	- infusion: 1–3 g of dried flowers per 250 mL of water, 3–4 times daily - extract: 250–500 mg, 1–3 times daily	moderate	strong	moderate	weak	[339,677,678]
Pot marigold (*Calendula officinalis*)	α-cadinol, rutin, quercetin, luteolin, lycopene, β-carotene, acidic pectic type polysaccharides, pectinoligosaccharides	flowers	- infusion: 1–2 g of dried flowers per 250 mL of water, 2–3 times daily - extract: 200–400 mg, twice daily (derived from commercial supplement product labels)	moderate	strong	moderate	weak	[348,679,680]
Ceylon cinnamon (*Cinnamomum verum*)	trans-cinnamaldehyde, eugenol	bark	- infusion: 0.5–1 g of powdered bark per 250 mL of water, 1–2 times daily - extract: 250–500 mg, 1–2 times daily	very strong	strong	strong	moderate	[447,681,682]
Dandelion (*Taraxacum officinale*)	taraxacin, fructooligosaccharides, luteolin, quercetin, chlorogenic acid, caffeic acid, taraxasterol	root, leaves	- infusion: 3–5 g of dried root per 250 mL of water, 2–3 times daily - extract: 250–500 mg of root extract, 2–3 times daily (derived from commercial supplement product labels)	strong	moderate	weak	very strong	[683,684]
Fennel (*Foeniculum vulgare*)	trans-anethole, fenchone, estragole, quercetin, rutin, kaempferol, dietary fibre, caffeic acid, quinic acid, chlorogenic acid, fructooligosaccharides	seeds (fruits)	- infusion: 1–3 g of crushed seeds per 250 mL of water, 2–3 times daily - extract: 200–400 mg, twice daily (derived from commercial supplement product labels)	moderate	moderate	moderate	weak	[519,685,686,687]
Garlic (*Allium sativum*)	alliin, allicin, ajoene, S-allyl cysteine, inulin, fructooligosaccharides	bulb (cloves)	- raw: 1–2 cloves daily - extract: 600–1200 mg of a standardised extract daily	strong	strong	very strong	strong	[444,522,567,688]
Ginger (*Zingiber officinale*)	α-tocopherol, ascorbic acid, [6]-gingerol, [6]-shogaol, zingiberene	rhizome	- infusion: 1–2 g of fresh or dried rhizome per 250 mL of water, 2–3 times daily - extract: 250–1000 mg daily in divided doses	strong	very strong	moderate	moderate	[443,444,689,690]
Green Tea (*Camellia sinensis*)	epigallocatechin gallate, epicatechin, epigallocatechin, epicatechin gallate, quercetin, kaempferol, gallic acid, caffeine	leaves	- infusion: 1–2 tsp (approx. 2.5–5 g) of leaves per 250 mL of water (80 °C), 2–3 times daily - extract: 250–500 mg of EGCG daily	very strong	strong	moderate	moderate	[657,691,692,693]

Note: Weak activity: denotes low intensity, a minor effect, or low strength. The effect is noticeable but minimal. Moderate activity: indicates a medium or average level. The activity is neither weak nor strong—it falls in the middle of the scale. It is clearly visible and measurable. Strong activity: describes high intensity, a significant impact, or considerable strength. The effect is distinct, potent, and easily observed. Very strong activity: represents an exceptional level of intensity, a primary therapeutic action, or a defining characteristic. The effect is robust and overwhelmingly supported by scientific evidence, often establishing the substance as a benchmark in its field.

## Data Availability

Data sharing is not applicable to this article.
